# Central Metabolism Is Tuned to the Availability of Oxygen in Developing Melon Fruit

**DOI:** 10.3389/fpls.2019.00594

**Published:** 2019-05-15

**Authors:** Kentaro Mori, Bertrand P. Beauvoit, Benoît Biais, Maxime Chabane, J. William Allwood, Catherine Deborde, Mickaël Maucourt, Royston Goodacre, Cécile Cabasson, Annick Moing, Dominique Rolin, Yves Gibon

**Affiliations:** ^1^UMR1332 BFP, INRA, Univ. Bordeaux, Villenave d’Ornon, France; ^2^Plateforme Métabolome Bordeaux, MetaboHUB, Bordeaux Functional Genomic Centre, Villenave d’Ornon, France; ^3^Environmental and Biochemical Sciences Group, The James Hutton Institute, Dundee, United Kingdom; ^4^Manchester Institute of Biotechnology, University of Manchester, Manchester, United Kingdom

**Keywords:** fruit, *Cucumis melo*, hypoxia, metabolism, modeling, cytochrome c oxidase

## Abstract

Respiration of bulky plant organs such as fleshy fruits depends on oxygen (O_2_) availability and often decreases with O_2_ concentration to avoid anoxia, but the relationship between O_2_ diffusional resistance and metabolic adjustments remains unclear. Melon fruit (*Cucumis melo* L.) was used to study relationships between O_2_ availability and metabolism in fleshy fruits. Enzyme activities, primary metabolites and O_2_ partial pressure were quantified from the periphery to the inner fruit mesocarp, at three stages of development. Hypoxia was gradually established during fruit development, but there was no strong oxygen gradient between the outer- and the inner mesocarp. These trends were confirmed by a mathematical modeling approach combining O_2_ diffusion equations and O_2_ demand estimates of the mesocarp tissue. A multivariate analysis of metabolites, enzyme activities, O_2_ demand and concentration reveals that metabolite gradients and enzyme capacities observed in melon fruits reflect continuous metabolic adjustments thus ensuring a timely maturation of the mesocarp. The present results suggest that the metabolic adjustments, especially the tuning of the capacity of cytochrome c oxidase (COX) to O_2_-availability that occurs during growth development, contribute to optimizing the O_2_-demand and avoiding the establishment of an O_2_ gradient within the flesh.

## Introduction

Bulky organs such as fruits, tubers or roots are prone to hypoxia and are known to decrease respiration with O_2_ concentration to avoid anoxia (Geigenberger, 2003). Their respiration usually follows Michealis-Menten kinetics but the involved mechanisms are still unclear (Ho et al., 2011). There is also the widespread idea that bulky organs are characterized by up to strong O_2_ gradients, as for example in potato tubers (Geigenberger, 2003). On the one hand, diffusional resistance in bulky organs has been invoked as explaining the occurrence of O_2_ gradients, which result in limited availability of O_2_ to respiration (Ho et al., 2011). On the other hand the recent finding of an O_2_ sensor in Arabidopsis (Licausi et al., 2011) suggests that plant tissues are capable of metabolic adjustments limiting respiration, which occur well above the K_m_ value for O_2_ of cytochrome c oxidase (COX). Strikingly, whereas the K_m_ value of COX is often invoked in literature dealing with hypoxia, its capacity is rarely taken into account, although its modulation is a further and obvious control point for respiration. The picture is further complicated by the fact that hypoxia is actually needed in a range of processes associated to ripening. Thus, the production of a range of volatiles involves alcohol dehydrogenases (ADHs) and further component of fermentation (Manriquez et al., 2006). Due to its large size and easily accessible flesh, melon (*Cucumis melo* L.) is an interesting model to study relationships between oxygen availability and metabolism as influencing fruit growth and quality. Indeed, the balance between respiration and fermentation is at the heart of fruit development and the establishment of their quality during ripening (Pesis, 2005). Besides, melon is an economically important crop with an expanding world production situated around 32 million tons in 2012^1^. It is a member of the Cucurbitaceae family, which represents a yearly world market of hundreds of billions of dollars and 50 kg per person, with watermelon alone being the second most produced fruit in the world^1^.

Whilst fruit flavor quality is largely the result of volatile compounds and their contribution to aroma, sweetness depends upon sugar concentration and the ratio between sugars and acids. Thus, melon fruits at commercial maturity are characterized by high contents of sucrose, glucose and fructose, as well as by a low organic acid content and the absence of starch (Lingle and Dunlap, 1987; Schaffer et al., 1987; Hubbard et al., 1989; Burger et al., 2000). The accumulation of these three soluble sugars appears to be controlled by carbohydrate metabolism in the fruit sink itself. Indeed, in the Curcubitaceae family, sucrose and the galactosyl-sucrose oligosaccharides, raffinose and stachyose, are synthetized in the leaf source and translocated to the fruit sink (Mitchell et al., 1992; Fiehn, 2003). Following which, raffinose and stachyose do not accumulate in the fruit, thus indicating a rapid metabolic conversion. Melon is a highly polymorphic species, in which variations in fruit sugar and acid contents are not just under genetic control, but are also influenced by the environment (Burger et al., 2000; Burger and Schaffer, 2007; Tang et al., 2012; Cohen et al., 2014). Numerous studies of fruit growth and ripening have shown that melon fruits undergo dramatic metabolic transitions. In particular, the accumulation of sucrose occurring at ripening has been associated with the loss of soluble acid invertase (AI) and a concomitant increase in sucrose phosphate synthase (SPS) (McCollum et al., 1988; Hubbard et al., 1989; Iwatsubo et al., 1992; Lester et al., 2001; Burger and Schaffer, 2007). However, both sucrose synthase (SuSy) and neutral invertase (NI) also show increased activities at ripening (Schaffer et al., 1987; Hubbard et al., 1989).

Recently, metabolomics approaches have been developed to identify key metabolites and their variations in fruits of various melon cultivars, including spatial and developmental measurements (Biais et al., 2009; Moing et al., 2011; Bernillon et al., 2013). Data analysis revealed several gradients of metabolites in fruit flesh at maturity such as sucrose, alanine, valine, γ-aminobutyric acid (GABA) and ethanol (Biais et al., 2010), which can be related with differences in metabolism. A decrease in the ATP/ADP ratio was also found to be associated with changes in alanine, GABA and ethanol, and was interpreted as being the result of acclimation to oxygen limitation as operated via alcoholic fermentation.

In the present study, metabolic changes occurring from the periphery to the fruit center and across three stages of fruit development were investigated using a multilevel approach. Thus major primary metabolites were determined by proton nuclear magnetic resonance spectroscopy (^1^H NMR) and gas chromatography coupled to mass spectrometry (GC-EI-TOF/MS) to investigate how and when metabolic gradients take place during melon fruit development. Enzyme activities of sucrose metabolism, tricarboxylic acid (TCA) cycle and glycolysis were measured using robotized assays and a microplate-based assay for COX capacity was developed to investigate its involvement in fruit central metabolism. Oxygen partial pressure was measured by using an oxygen-sensitive optical glass-sensor and a model was built to calculate steady state O_2_ concentrations at any position within the mesocarp based on O_2_ diffusion and consumption. The calculation of O_2_ demand using a construction cost model took in account the rates of biomass production at different depths within the mesocarp. By combining these approaches we investigated the relationship between O_2_ diffusional resistance and metabolic adjustments occurring in melon fruit development.

## Materials and Methods

### Melon Growth and Sample Handling

**FIGURE 1:**
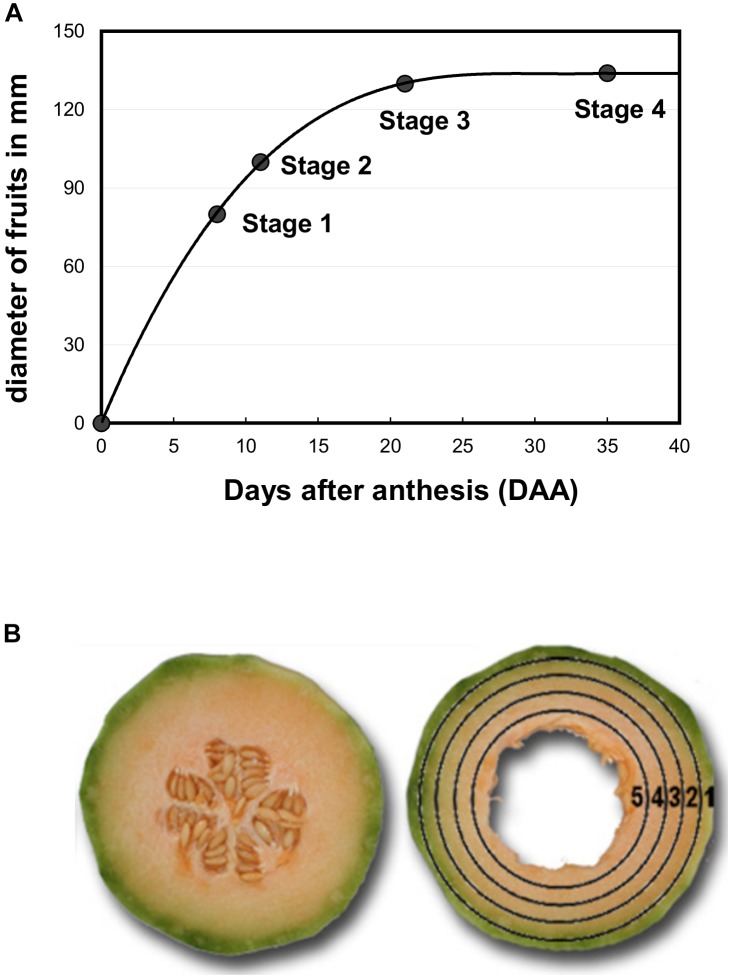
**(A)** Growth curve of melon fruits (*Cucumis melo* L. var Cantalupensis group Charentais cv. Escrito). The diameter of harvested fruits was measured at each stage of development and was used to assess the growth curve. Melon fruits at stages 1, 2, 3, and 4 correspond to fruits harvested at 8, 11, 20, and 34 days after anthesis (DAA). **(B)** Sample preparation for biochemical analyses. A slice (7 ± 2 mm width) was first cut at the equator of the fruit. The seeds and placenta were removed, and then five concentric rings were cut in the slice and immediately frozen in liquid nitrogen. Position 1 corresponds to both the epicarp and green mesocarp, and positions 2–5 to the orange mesocarp.

Melon plants (*Cucumis melo* L. var. Cantalupensis group Charentais cv. Escrito) were grown in an open field (9200 plants ha^−1^) in Moissac (France, 44° 6′ 17″ N, 1° 5′ 6″ E) between April and August 2008 and Sainte-Livrade (France, 44° 23′ 56″ N, 0° 35′ 25″ E) between May and September 2011. Irrigation, fertilization and pathogen-pest control were performed according to standard commercial practices (Bernillon et al., 2013). Melon fruits were harvested at four stages of development (Figure 1A). Stage 1 corresponds to developing fruits with a diameter of 80 mm, Stage 2 to developing fruits with a diameter of 100–110 mm just before the appearance of the suberized net on the skin, Stage 3 to early ripening fruits with a fruit diameter of 130–135 mm and Stage 4 to ripening fruits at the beginning of abscission (commercial maturity). For O_2_ tension measurements in melon mesocarp, 32 melons were harvested from Stages 1–3 and kept at laboratory temperature during the measurements. For ^1^H-NMR analysis of polar compounds and enzyme assays, stages 2–4 were used. Nine melons were selected to make three homogeneous lots (biological replicates) of three fruits each. Two slices of 1 cm thickness were cut in the equatorial plane of each fruit. The skin and seeds were removed and five concentric mesocarp rings of flesh (7 ± 2 mm width) were taken from the periphery (outer mesocarp, named location 1) to the centre (inner mescocarp, named location 5) as explained in Biais et al. (2010) and Figure 1B. The flesh rings of a given position taken from a given fruit lot were pooled and immediately frozen in liquid nitrogen, before then being stored at −80°C. These were subsequently ground in liquid nitrogen and the 45 powdered samples (three stages × three biological replicates × five flesh positions) were further stored at −80°C until further processing. For ^1^H-NMR analysis an aliquot of each sample was lyophilized. In parallel, dry matter content was determined using 250 mg FW powder aliquots dried in a 70°C oven.

### Chemicals

All chemicals and substrates used for chemical and biochemical analyses were purchased from Sigma-Aldrich Ltd. (Gillingham, United Kingdom), except for acetyl coenzyme A, adenosine-5′-triphosphate (ATP), dithiothreitol, *n*-dodecyl β-D maltopyranoside, leupeptin, nicotinamide adenine dinucleotide (NAD), NADH, nicotinamide adenine dinucleotide phosphate (NADP), NADPH and phosphoenolpyruvate, that were purchased from Roche Applied Science (Meylan, F). All enzymes were purchased from Roche Applied Science (Meylan, F) except aldolase (from rabbit muscle), glycerokinase (from *E. coli*) and triose phosphate isomerase (from rabbit muscle) that were purchased from Sigma-Aldrich Ltd. (Gillingham, United Kingdom). Bradford reagent was purchased from Bio-Rad (Marnes-la-Coquette, F). Reagents used for mRNA quantification were purchased from Fisher Scientific (Illkirch, F), Qiagen (Courtaboeuf, F), Bio-Rad (Mitry-Mory, F), and Promega (Charbonnières-les-Bains, F).

### ^1^H NMR and GC-EI-TOF/MS Analyses of Polar Metabolites of Ground Flesh Samples

Polar metabolites were extracted from ground melon samples corresponding to the five concentric mesocarp rings of flesh. For NMR analyses the frozen powdered samples were lyophilized and polar metabolites were extracted from 50 mg of lyophilized powder successively with 2 ml of ethanol/water mixtures: 80/20, 50/50 (v/v) and pure water (4 ml) for 15 min at 80°C. The supernatants were combined, dried under vacuum and lyophilized. The pellet was kept for the determination of protein content. Two technical replicates were prepared per biological sample. The lyophilized extracts were mixed with 500 μl of 400 mM potassium phosphate buffer solution pH 6.0, 1 mM ethylene diamine tetraacetic acid disodium salt (EDTA), in D_2_O. The pH was the adjusted to 6 with KOD when necessary, and lyophilized again. The lyophilized extracts were stored in darkness under vacuum at room temperature, before ^1^H-NMR analysis which was completed within 1 week. For ^1^H-NMR analysis, each dried pH-adjusted extract was solubilized in 0.5 ml of D_2_O with (trimethylsilyl) propionic-2,2,3,3-d4 acid (TSP) sodium salt (0.01% final concentration for chemical shift calibration), centrifuged at 10,000 g for 5 min and transferred into an 5 mm NMR tube. Quantitative ^1^H-NMR spectra were recorded at 500.162 MHz and 300 K on a Bruker Avance spectrometer (Wissembourg, France), using a 5 mm inverse probe and an electronic reference for quantification as described previously (Mounet et al., 2007; Biais et al., 2009; Moing et al., 2011). For GC-EI-TOF/MS, 100 mg aliquots ( ± 2 mg) of frozen ground samples were extracted with 1 ml chloroform/methanol/water (1:2.5:1), the polar metabolite fraction was obtained by further addition of 0.5 ml of water and dried by speed vacuum concentration. The extracts were derivatized and analyzed by GC-EI-TOF/MS on an Agilent 6890N gas chromatograph (Stockport, United Kingdom), coupled to a Leco Pegasus III mass spectrometer (St Joseph, United States). Chromatographic deconvolution was performed within the LECO ChromaTof v2.15 software, the extracted peak areas for each of the deconvolved metabolite features were normalized against the succinic-*d4* acid internal standard. The detailed procedures for extraction, derivatization, sample analysis, mass spectral deconvolution and metabolite identification, have been described in detail previously (Allwood et al., 2009; Biais et al., 2009; Moing et al., 2011). The full dataset can be found at http://www.cbib.u-bordeaux2.fr/MERYB/public/PublicREF.php?REF=M08002.

### Protein Content

The pellet was resuspended in 1 ml 100 mM NaOH and heated for 30 min at 95°C. After cooling to room temperature and centrifugation at 5,000 g, the protein content of the remaining supernatant was determined according to Bradford (1976).

### Enzyme Activity Measurements

Aliquots of 50 mg fresh weight (FW) were extracted by vigorous shaking with 500 μl of extraction buffer composed of 20% (v/v) glycerol, 0.25% (w/v) bovine serum albumin, 1% (v/v) Triton-X100, 50 mM HEPES/KOH pH 7.5, 10 mM MgCl_2_, 1 mM EDTA, 1 mM EGTA, 1 mM ε-aminocaproic acid, 1 mM benzamidine, 10 mM leupeptin, 0.5 mM dithiothreitol, and 1 mM phenylmethylsulfonylfluoride, which was added just prior to extraction. The following enzyme activities (SPS, SUSY, acid invertase, PFP, PFK, MDH, PK, PEPC, CS, IDH, see Figure 3 legend for the full enzyme name) were assayed using a robotized platform as described in Gibon et al. (2004, 2006, 2009), Studart-Guimaräes et al. (2005), and Steinhauser et al. (2010).

Enolase was assayed in the direction of phospho*enol*pyruvate production as described previously (Burell et al., 1994). The assay consisted of 10 μl desalted extract in 100 mM HEPES/NaOH (pH 7.5), 10 mM MgCl_2_, 0.2 mM NADH, 2.7 mM ADP, 5 units.ml^−1^ pyruvate kinase and 6 units.ml^−1^ lactate dehydrogenase. The reaction was initiated by the addition of 2-phosphoglycerate to a final concentration of 0.5 mM.

NADP dependent malic enzyme activity (NADP-ME) was assayed using a protocol adapted from Wheeler et al. (2005). The assay was performed with 2 μl of extract in 100 mM HEPES/KOH (pH 7.5), 10 mM MgCl_2_, 1 mM NADP^+^ and 0.05% (v/v) Triton X100. The reaction was initiated by addition of malate to a final concentration of 10 mM and a final volume of 20 μl. After 40 min of incubation at 25°C, the reaction was stopped by the addition of 20 μl of HCl 0.5 M/Tricine/KOH 0.1 M (pH 9). Following sample mixing and a 10 min delay, the acid was neutralized by the addition of 20 μl of 0.5 M NaOH. The quantification of phospho*enol*pyruvate was then performed by adding 100 mM HEPES/KOH (pH 7.5), 10 mM MgCl_2_, 0.05% (v/v) Triton X100 and 1 mM NADH in a final volume of 110 μl. Absorbance at 340 nm was recorded until stabilized, then 2 μl of lactate dehydrogenase 100 units.ml^−1^ were added to start the reaction and absorbance was again recorded until stabilized.

Alcohol dehydrogenase (ADH) activity was assayed using 5 μl of extract in 100 mM HEPES/KOH (pH 7.5), 0.1 mM EDTA, 1.2 mM NAD^+^, 1 mM thiazolyl blue tetrazolium bromide (MTT), 0.2 mM phenazine ethosulfate and 0.05% (v/v) Triton X100. The reaction was initiated by the addition of ethanol to a final concentration of 25 mM and a final volume of 100 μl. Absorbance was read at 600 nm until rate stabilized.

The respiratory chain enzymes, i.e., cytochrome *c* oxidase (COX) and succinate dehydrogenase (SDH), were assayed as described in Rustin et al. (1994) and Vinogradov et al. (1980), respectively, using a modified extraction procedure adapted to plant tissues. Briefly, aliquots of 100 mg FW powder were extracted at 4°C by vortexing with 500 μL of potassium phosphate buffer solution (50 mM potassium phosphate pH 7.2, 1 mM EDTA) supplemented with 1% (w/v) *n*-dodecyl β-D maltopyranoside. To eliminate low molecular weight reducing compounds and to decrease the detergent concentration, the homogenate was subsequently filtrated through a 2 ml column of Sephadex G25 coarse medium (Sigma-Aldrich, Lyon, France), equilibrated at 4°C with the phosphate buffer solution supplemented with 0.1% (w/v) *n*-dodecyl β-D maltopyranoside. Sephadex columns were then centrifuged at 4°C for 2 min at 2400 *g* and the filtrate stored on ice. The enzyme recovery was checked using isolated mitochondria for which COX and SDH activities can be measured before and after filtration. SDH was assayed at 25°C using the phenazine methosulfate- (PMS) mediated reduction of dichloroindolphosphate (DCIP) monitored at 600 nm with a spectrophotometer. The SDH assay consisted of 10–50 μL of filtrate in a final volume of 1 ml of phosphate buffer solution (pH 7.2) containing 0.1% (w/v) *n*-dodecyl β-D maltopyranoside, 5 mM succinate, 0.1 mM KCN and 0.05 mM DCIP. The reaction was initiated by the addition of PMS to a final concentration of 1.6 mM. COX was assayed at 25°C by monitoring the oxidation of reduced cytochrome *c* at 550 nm. The COX assay consisted of 10–50 μL of filtrate in a final volume of 1 ml of phosphate buffer solution (pH 7.2) containing 0.1% (w/v) *n*-dodecyl β-D maltopyranoside. The reaction was initiated by the addition of chemically-reduced cytochrome *c* at a final concentration of 50 μM. The specificity of the assay was checked by addition of KCN.

### O_2_ Measurements Within the Melon Mesocarp

The O_2_ tension (expressed in kPa) was measured by using an oxygen-sensitive optical glass-sensor (microsensor, PreSens, Neuburg, DE) connected to a fiber optic oxygen meter (MicroX TX3 PreSens, Neuburg, DE), and was based upon dynamic fluorescence quenching. The O_2_ microprobe tip has a diameter of 140 μm and is protected by a hypodermic needle linked to a syringe. In contrast to oxygen microelectrodes, this oxygen microprobe does not consume oxygen and shows no stirring dependence of the signal, and thus prevents the establishment of an artificial oxygen sink within the measured tissue. The microsensor was calibrated in water that had been well equilibrated with ambient air (21 kPa O_2_) and also in water that had been depleted of oxygen with Na_2_SO_3_. The electrode signal was stable for at least 4 h. The fruit was first placed on a support and fixed. Subsequently, the microsensor was positioned on the fruit surface and driven into the fruit by a micromanipulator at 5 mm intervals. Just after the insertion of the needle, the microsensor occupied the small hole in the tissue at the needles entry point, without being in contact with the outside. At each position the sensor was paused for approximately 120 s to allow equilibration and to obtain a continuous measurement. The mean of 100 measurements with a standard error of less than 5% represents a single data point in the subsequently presented figures. From 32 melons harvested at stages 1–4, 16 were used within 48 h to measure O_2_ tension at three different depths (7.5, 12.5, and 17.5 mm) in the mesocarp and at three different equatorial positions. Only in mature fruits (stage 4), the O_2_ tension was measured deeper into the mesocarp (22.5, 27.5, and 32.5 mm). After the measurements were performed, the melon fruits were sliced at the level of the measurement transect to verify the exact position of the sensor tip within the distinct zones of the mesocarp.

### Modeling of the Oxygen Gradient Within the Melon Mesocarp

The oxygen concentration within the melon was modeled assuming that the steady state oxygen concentration at any position within the mesocarp is a function of the oxygen diffusion and consumption within the tissue. This was achieved by solving the time-dependent diffusion equation in spherical co-ordinates with spherical symmetry (McElwain, 1978):

$$\frac{\partial C}{\partial t}=D\left(\frac{\partial^{2}C}{\partial R^{2}}+\frac{2}{R}\frac{\partial C}{\partial R}-\frac{\alpha C}{C+K_{\text{m}}}\right)$$

with the following dimension-less variables:

$$C=\frac{P}{P_{0}};\;R=\frac{r}{r_{0}};\text{ α}=\frac{V_{\text{oxygen}}r_{0}^{2}}{P_{0}D};\;K_{\text{m}}=\frac{k_{\text{m}}}{P_{0}}$$

Where: *R* is the relative distance from the centre, *r*_0,_ the radius of the melon, D, the diffusion coefficient within the mesocarp (*D* = 2.4 10^−5^ cm^2^ s^−1^) and *P*_0_, the oxygen tension outside the melon (21 kPa, at 25°C). *K*_m_ is the half saturation constant of the terminal oxidase of the respiratory chain. It was equal to either 0.108 or 0.134 kPa, for the COX or the alternative oxidase, respectively (Armstrong and Beckett, 2011). These values were assumed to be constant as a function of the depth (i.e., as a function of the respiratory rate). *V*_oxygen_ is the O_2_ consumption rate of the tissue, which was estimated using a construction cost model (see below) and was computed as a depth-dependent polynomial function (see Supplemental Figures S1, S2).

The steady state problem (∂*C*/∂*t* = 0) was solved using the PDEX4 function in Matlab 2007 and the following initial and boundary conditions:

at t=0, C=0

$$\begin{array}{cc} \text{at }r=0.5\;r_{0}, & \frac{\mathrm{dC}}{\mathrm{dR}}=0 \end{array}$$

$$\begin{array}{cc} \text{at }r=r_{0}, & \frac{\mathrm{dC}}{\mathrm{dR}}=\frac{{\mathrm{hr}}_{0}}{D}(1-C) \end{array}$$

where *h* is the permeability coefficient of the melon skin, expressed in cm s^−1^.

The Matlab script is provided within the Supplementary Material (Note 1 in Supplemental Data).

For each developmental stage, an *h*-value was obtained by fitting the O_2_ measurements and minimizing an *Obj* score, i.e., the sum of the squared residuals weighed by the standard deviation of each measurement, according to the following equation:

$$\mathrm{Obj}=\sum_{\text{i}=1}^{n}{\left(\frac{{\left[{\text{O}}_{2}\right]}_{\text{i}}\mathrm{cal}-{\left[{\text{O}}_{2}\right]}_{\text{i}}\exp}{{\text{σ}}_{\text{i}}\exp}\right)}^{2}$$

where *n* is the number of depths, *[O*_2_*]*_i_*cal*, and *[O*_2_*]*_i_*exp*, the calculated and experimental values of O_2_ concentration at a given depth and σ_i_*exp*, the standard deviation of the measures.

### Calculation of the Oxygen Demand Using a Construction Cost Model

Rates of biomass production at different depths within the mesocarp were calculated by expressing biomass as a function of time for five layers of equal thickness. For this, considering that the fruit is a sphere, the volumes of the six corresponding layers nested into one another’s spheres, were calculated throughout development, based on estimates of the changes in fruit diameter, and subtracted to obtain the volumes of the sectors (see Supplemental Figures S1, S2). Volumes were then converted to biomass using density estimates and fitted polynomials that were integrated to obtain the rates of biomass production.

The flux of oxygen consumption was calculated at each stage and for each sector as the sum of the growth-linked and maintenance respiration, according to the following equation (Heuvelink, 1995; Liu et al., 2007):

(1) $$\frac{{\mathrm{dC}}_{\text{respiration}}}{\mathrm{dt}}=q_{\text{growth}}*\frac{\mathrm{dDW}}{\mathrm{dt}}+q_{\text{maintenance}}*\mathrm{DW}*Q_{10}^{(t{}^{\circ}-20)/10}$$

where: *dC*_respiration_/*dt* is the respiration-linked carbon consumption of the sector (g of C day^−1^ g^−1^ *DW*); *dDW*/*dt* and *DW*, the growth rate and the dry weight content of the sector, respectively, *Q*_10_, the temperature-dependent coefficient for maintenance respiration (*Q*_10_ = 2) and *t*°, the growth temperature (averaged temperature of the culture was 25.4°C). *q*_growth,_ the carbon requirement for growth-linked respiration, is well known for a variety of fleshy fruits and is equal to 0.1 g C g^−1^ DW at 20°C (Heuvelink, 1995; Liu et al., 2007; Dai et al., 2010, and references therein). In contrast, *q*_maintenance_, the carbon requirement for cell maintenance, is hardly available for fruits and needs to be estimated. It was calculated assuming that the maintenance-linked respiration of aerobically grown cells is proportional to the amount of respiratory complexes and accounts for 10–20% of the complex IV (i.e., COX) activity (Devin et al., 2006, and references therein). Finally, the oxygen consumption rate (expressed in μmol O_2_ min^−1^.ml^−1^) was calculated by using the sector volume, the fresh-to-dry weight ratio, assuming that the tissue density is equal to 1 g FW per ml and considering that the coefficient of respiration equals 1, i.e., neglecting the ethanol production vs. oxidative phosphorylation.

### Total RNA Extraction and qRT-PCR Analysis

Total RNA was extracted from melon mesocarp tissues using Trizol reagent (Fisher scientific). One hundred milligrams of frozen mesocarp powder were homogenized with 1 ml of Trizol reagent and spiked with a mix of the five artificial poly(A+) RNAs (ArrayControl^TM^ Spots and Spikes, Fisher Scientific) to achieve absolute quantification of transcripts. Concentration of RNA spikes were 2.4 × 10^11^, 4.8 × 10^10^, 2.4 × 10^9^, 2.5 × 10^7^, and 4.0 × 10^6^ copies per gram FW. Total RNA was purified with the RNeasy kit (Qiagen), then treated with the Turbo DNase (Fisher Scientific). cDNA was synthesized from 500 ng total RNA using the iScript cDNA synthesis kit (Bio-Rad) and was diluted 5 times with nuclease free water. qRT-PCR reactions and data analysis were performed as described by Piques et al. (2009) with slight modifications. The 20 μl PCR mixture contained 2 μl of the diluted cDNA template, 10 μl of 2 × GoTaq^®^ qPCR Master Mix (Promega), and 0.2 μM of the forward and reverse primers for each gene. The primers used to amplify melon genes were designed using primer3 software^2^. The primers for the RNA spike-in controls were designed as described in Pyl et al. (2012). All primer sequences are available in Supplemental Table S2. PCR reactions were run on the CFX96^TM^ Real-Time PCR Detection System (Bio-Rad) under the following conditions: 95°C for 2 min, followed by 40 cycles at 95°C for 3 s, at the annealing temperature of 60°C for 30 s, and a dissociation curve analysis, 65–95°C with temperature increment of 0.5°C every 5 s. For each sample, cycle threshold values and copy numbers for the 5 spike-in controls were used to generate a standard curve. All standard curves, derived from the five spike-in controls, had *R*^2^-values higher than 0.99 and were used to calculate the concentration of the mRNA as copy number g^−1^ FW and copy number mg^−1^ protein.

### Statistical Analysis

Principal component analysis (PCA) and correlation analysis (Pearson coefficient) were performed using R^3^ with the package FactomineR (Lê et al., 2008).

## Results

Melon fruits (*Cucumis melo* var. Cantalupensis group Charentais cv. Escrito) were grown in the open field under common agricultural practices; the fruits were ripe within 45 days post anthesis. The Escrito cultivar, which is widely cultivated in France, is considered as average regarding aromaticity and shelf-life (Allwood et al., 2014). To study the metabolic changes that occur during ripening, samples were taken during fruit growth (stage 2), at early fruit ripening (stage 3) and at fruit maturity (stage 4) (Figure 1A). For each stage, nine fruits were harvested and cut into five concentric mesocarp rings, the concentric tissue sections were pooled for three fruits and homogenized, thus creating three replicate samples each made up of three pooled fruits, prior to extraction and metabolite quantification (Figure 1B). For oxygen pressure measurements, an additional stage corresponding to fruit growth (stage 1) was used because data obtained at stage 2 appeared more scattered than at stages 3 and 4.

### Maturation of the Melon Mesocarp Is Centrifugal

**FIGURE 2:**
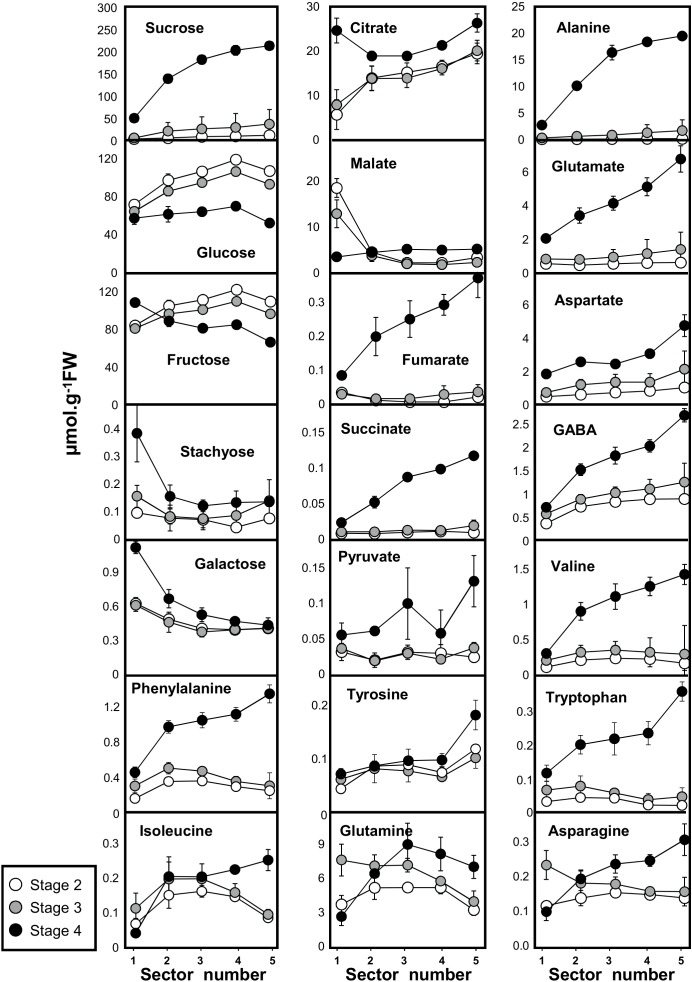
Absolute concentrations in 18 primary metabolites measured by quantitative ^1^H NMR spectroscopy (16 metabolites) and GC-EI-TOF/MS (pyruvate and succinate) in developing melon (*Cucumis melo* L. var Cantalupensis group Charentais cv. Escrito) fruit harvested at three developmental stages. Sector 1, 2, 3, 4, and 5 correspond to five concentric mesocarp rings taken from the periphery (outer epicarp + green mesocarp, named sector 1) to the fruit centre (inner mescocarp, named sector 5). Concentrations are given in μmol.g^−1^FW. The results are the means of 9 measurements (3 biological replicates × 3 technical replicates), bars represent standard error (*n* = 3). The data are available in Supplemental Table S1.

The changes in metabolites that occurred during fruit development in the five concentric mesocarp rings of flesh taken from the periphery (outer mesocarp, sector 1) to the centre (inner mesocarp, sector 5) are presented in Figure 2 (see also Supplemental Table S1). Soluble sugars were the most abundant metabolites throughout fruit development. Whilst sugars such as stachyose and galactose exhibited a steady and very low content in the fleshy mesocarp at the three development stages (0.15 and 0.5 μmol.g^−1^ FW, respectively), Suc, glucose (Glc), and fructose (Fru) (the major soluble sugars in melon) revealed different patterns. Suc exhibited a steady and relatively low content in the fleshy mesocarp around 20–40 μmol.g^−1^ FW during fruit development (stage 2) and early fruit ripening (stage 3), whilst at fruit maturity (stage 4) a strong Suc gradient was observed from the periphery to the center of the fruit. Suc concentration increased regularly from the periphery (50 μmol.g^−1^ FW, position 1) to the center of the fruit (200 μmol.g^−1^ FW, position 5). In contrast, Glc and Fru concentrations were highest during fruit development, ranging between 80 and 110 μmol.g^−1^ FW at stages 2 and 3 and decreased at fruit maturity (stage 4), with 60 and 80 μmol.g^−1^ FW for Glc and Fru, respectively. The two hexoses exhibited a small gradient between outer and inner mesocarp at stages 2 and 3. The Glc and Fru concentrations increased regularly from the periphery (80 μmol.g^−1^ FW, position 1) to the center of the fruit (110 μmol.g^−1^ FW, position 4 and 5). At maturity the Glc gradient disappeared and the Fru gradient was inverted.

Among the five detected organic acids, pyruvate, fumarate and succinate showed the same pattern. During fruit development (stage 2) and at the early ripening stage (stage 3), they were almost undetectable, but increased dramatically at fruit maturity, where a marked gradient was also observed from the periphery to the center of the fruit. Malate and citrate had different patterns. With the exception of the outer mesocarp (position 1), malate levels remained low and stable throughout the rest of the fleshy mesocarp (less than 5 μmol.g^−1^ FW), whilst a small gradient was apparent for citrate between the outer and inner mesocarp (15–20 μmol.g^−1^ FW) during stages 2 and 3. At maturity, the citrate gradient was still apparent in the fleshy sectors, with slightly higher concentrations (20–25 μmol.g^−1^ FW) than observed during the earlier developmental stages, further a strong increase of citrate was observed in the peripheral sector at full maturity.

Eleven and nine amino acids were measured by GC-EI-TOF/MS and ^1^H-NMR, respectively. Among the eight major amino acids observed (Figure 2), five were found at concentrations greater than 2 μmol.g^−1^ FW in the fleshy mesocarp at maturity (Ala, Gln, Glu, Asp, and GABA). All these amino acids, as well as the three aromatic amino acids, showed the same pattern of accumulation. Whilst no strong gradients were observed for amino acids during development, marked gradients were observed from the periphery to the center of the fruit for almost all amino acids measured at full maturity in this study. Interestingly, the fact that at ripening the protein content did not decrease while amino acids were strongly increased indicates that the latter increase was not due to proteolysis (Supplemental Figure S3). In conclusion, when comparing the metabolic shifts that occur throughout the different developmental stages of the melon fruit, characteristic changes in patterns of metabolite accumulation were revealed to be common between amino acids, organic acids and Suc. The gradients observed at different time points and from the periphery to the center of the fruit for amino acids (Ala, Val, Glu, GABA, Asp, Asn, Ile, Phe, Tyr, Trp), organic acids (succinate, fumarate, pyruvate, and citrate) and Suc indicates that the process of maturation starts from the inner part of the fruit. We next therefore investigated whether these metabolic changes were associated with changes in the activities of enzymes involved in central metabolism.

### Most Enzyme Capacities Show Marked Spatial and/or Developmental Gradients

**FIGURE 3:**
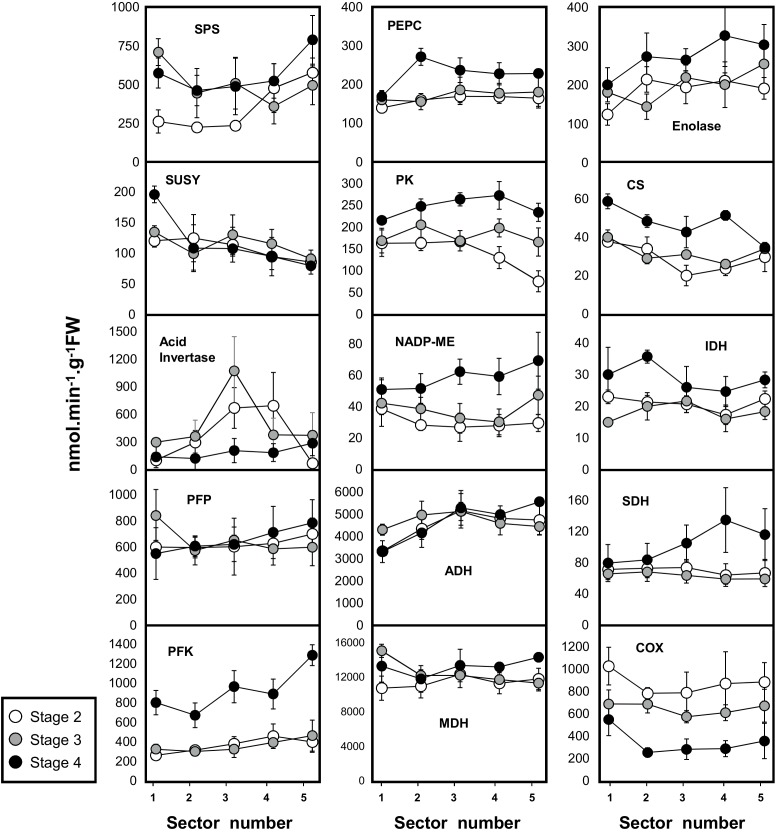
Activities of 15 enzymes from central metabolism during the development of melon (*Cucumis melo* L. var Cantalupensis group Charentais cv. Escrito) fruit harvested at 3 developmental stages. Sector 1, 2, 3, 4, and 5 correspond to five concentric mesocarp rings taken from the periphery (outer epicarp + green mesocarp, named sector 1) to the fruit center (inner mescocarp, named sector 5). Three enzymes involved in sugar metabolism [sucrose synthase (SUSY), acid invertase, sucrose phosphate synthase (SPS)], enolase, 4 glycolytic enzymes [ATP-dependent phosphofructokinase (PFK), pyrophosphate dependent phosphofructokinase (PFP), pyruvate kinase (PK)], 4 enzymes of the tricarboxylic acid cycle [NAD-dependent malate dehydrogenase (MDH), isocitrate dehydrogenase (IDH), citrate synthase (CS), succinate dehydrogenase (SDH)], 2 anaplerotic enzymes [phosphoenolpyruvate carboxylase (PEPC) and NADP malic enzyme (NADP-ME)], 1 enzyme of the respiratory chain [cytochrome c oxidase (COX)] and 1 enzyme involved in fermentation [alcohol dehydrogenase (ADH)]. Enzyme activities are expressed as nmol.g^−1^FW.min^−1^. The results are the means of 3 × 3 measurements (3 biological replicates × 3 technical replicates), bars represent standard error (*n* = 3). The data are available in Supplemental Table S1.

In order to follow metabolic changes in mesocarp tissue during melon fruit development, we profiled capacities (i.e., maximal measurable catalytic activities) of 15 enzymes from central metabolism (Figure 3, see also Supplemental Table S1). The developmental pattern of Suc accumulation at fruit maturity (stage 4) and the concomitant decrease in Glc and Fru contents were associated to a reduction in acid invertase activity (600–187 nmol.min^−1^. g^−1^ FW, average of the whole mesocarp at stages 2 and 4). The clear pattern observed for acid invertase was not consistently observed for the two other enzymes involved in Suc metabolism, i.e., SPS and SuSy. A moderate increase in SPS at fruit maturity (stage 4) compared to developing fruit (stage 2) (356–568 nmol.min^−1^. g^−1^ FW, average of the whole mesocarp at stages 2 and 4) was observed while for SuSy there was a small gradient between the outer and inner mesocarp (sectors 1–5) at all developmental stages (196–80 nmol.min^−1^. g^−1^ FW, stage 4) (Figure 3).

We next explored variations in the capacities of glycolytic enzymes such as ATP-dependent phosphofructokinase (PFK), PPi-dependent phosphofructokinase (PFP), enolase and pyruvate kinase (PK). These four glycolytic enzymes exhibited comparable profiles throughout fruit development. Their activities remained low during stages 2 and 3 while their highest activities were observed at fruit maturity (stage 4). For the four glycolytic enzymes (PFK, PFP, enolase, PK), a net gradient could be observed between sector 1, which corresponds to the green mesocarp below the epicarp, and sectors 2–5, which correspond to the orange mesocarp at full maturity.

Next, the activities of TCA-cycle enzymes, i.e., citrate synthase (CS), NAD-isocitrate dehydrogenase (IDH), SDH and NAD-malate dehydrogenase (MDH), as well as two further enzymes involved in the metabolism of organic acids, i.e., phospho*enol*pyruvate carboxylase (PEPC) and NADP-malic enzyme (ME), were investigated (Figure 3). These six activities had comparable profiles during the development of the melon fruit, and peaked at maturity (stage 4). No strong gradient could be observed for these enzymes involved in TCA and organic acid metabolism between the outer and inner mesocarp (except for CS). Conversely, the COX activity of the respiratory chain was relatively high in the mesocarp during stage 1 (871 nmol.min^−1^. g^−1^ FW average for the whole mesocarp) and, regardless of the location within the tissue, decreased throughout development (down to 346 nmol.min^−1^. g^−1^ FW average for the whole mesocarp at maturity). A gradient of the COX capacity was also observed between sector 1 corresponding to the green mesocarp, and the orange sectors (from 549 to 259 nmol.min^−1^.g^−1^ FW at stage 4) (Figure 3). Considering that fermentation is particularly important in fruit, ADH was also investigated. ADH activity was high at all three developmental stages and surprisingly high in the external layer of the mesocarp where it reached 3,320 nmol.min^−1^.g^−1^ FW at stage 2. At maturity, ADH showed the same gradient pattern as the activities of the glycolytic enzymes (PFP, PFK, Enolase, and PK; Figure 3).

To summarize, among the 15 tested enzyme activities expressed on a FW basis, only the COX activity of the respiratory chain and acid invertase activities showed a clear decrease at maturity while the other enzymes were stable or tended to slightly increase. The intriguing results obtained with enzymes involved in fermentation (high ADH activity at the periphery and before ripening) and respiration (strong decrease of the COX activity at maturity) prompted us to investigate O_2_ availability, as lowering oxygen within the mesocarp might lead to hypoxia and could influence the energy status, which can affect primary metabolism.

### Hypoxia Is Gradually Established During the Development of the Melon Fruit Although There Is No Strong Oxygen Gradient Between the Outer- and the Inner Mesocarp

**FIGURE 4:**
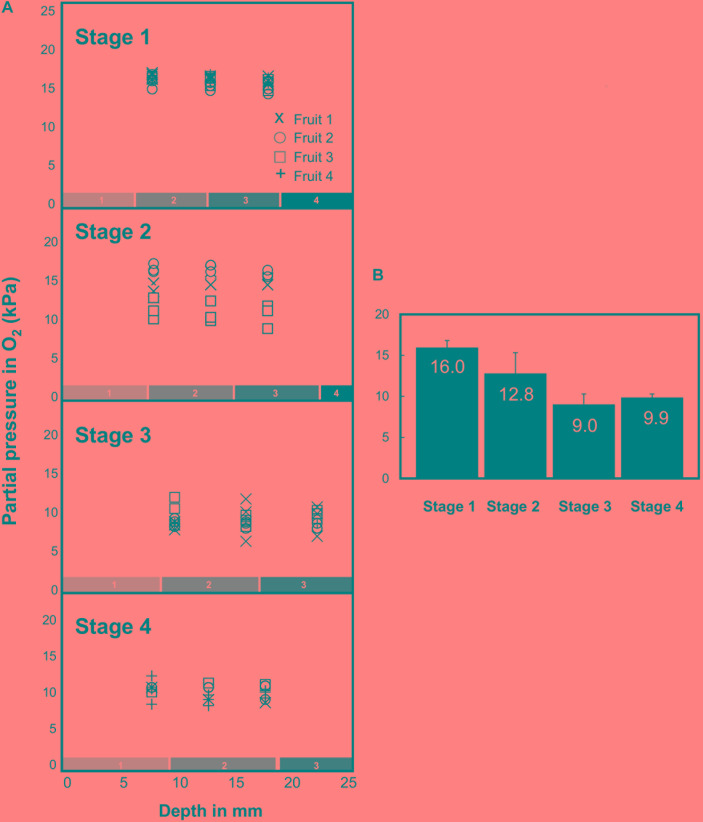
Oxygen tension (expressed in kPa) measured in the mesocarp of 16 melon (*Cucumis melo* L. var Cantalupensis group Charentais cv. Escrito) fruits harvested at stages 1, 2, 3, and 4 with an oxygen-sensitive optical glass-sensor connected to a fiber optic oxygen meter (MicroX TX3 PreSens) based on dynamic fluorescence quenching. **(A)** O_2_ tension measured at 3 depths (7.5, 12.5, and 17.5 mm) in the mesocarp and at three different equatorial positions. In mature fruits (stage 4), O_2_ tension was measured deeper in the mesocarp (22.5, 27.5, and 32.5 mm). The numbered boxes (gray to black, 1–4) indicate the evolution of the size of the harvested concentric mesocarp ring at stages 1–4. **(B)** Mean O_2_ tensions measured in melon fruits harvested at developmental stages 1–4.

Fine glass microsensors (tip diameter 140 μm) were used to measure O_2_ concentrations at three to five different depths in the mesocarp (from 7.5 to 32.5 mm) from three different equatorial positions of fruits harvested across the developmental stages 1–4. The microsensor was introduced into the mesocarp on the equator axis and was driven toward the center of the fruit (Figure 4A). Regardless of the developmental status, the O_2_ level immediately declined just below the external layer of the mesocarp, from 21 kPa (atmospheric O_2_ level) to 16.3 kPa at stage 1 or even to 10.5 kPa at stage 4 (Figure 4A). Then, within fruits harvested at stage 1 (developing fruits with a diameter of 80–90 mm), O_2_ decreased slightly from 16.3 kPa in the outer layer to 15.5 kPa at 17.5 mm depth. At this developmental stage, all investigated fruits showed similar O_2_ profiles. In developing fruits with a diameter of 100–110 mm that are characterized by the absence of the suberized net on the skin (stage 2), the O_2_ concentration seemed to be stable regardless of the position on the fruit where it was recorded, but there was a relatively large variability between fruits. Indeed, some fruits at stage 2 showed comparable O_2_ concentrations to fruits at stage 1 (around 16.3 kPa), while others showed lower O_2_ concentrations (down to 15.5 kPa). These results suggest that fruits selected on the basis of their diameter for stage 2 can in fact be at different developmental stages. The lowest O_2_ levels were always detected in early ripening fruits (stage 3), i.e., in fruits with diameter of 130–135 mm (down to 9 kPa). In mature fruit (stage 4), the O_2_ concentration was very stable across all tissue sections and types (9.9 kPa). The results show that in the mesocarp O_2_ concentrations decrease throughout fruit development and that the O_2_ concentration was consistent throughout the whole mesocarp regardless of tissue depth. The relationship between mean O_2_ tensions measured in melon fruits harvested at different developmental stages is given in Figure 4B. A steady decrease in O_2_ was found during the development of melon fruit from stages 1 to 3 and the O_2_ tensions remained very stable at around 9.9 ± 0.4 kPa in the whole mesocarp without there being an apparent O_2_ gradient in the tissue.

Given the intriguing absence of marked gradients in the oxygen concentrations of the mesocarp, it was logical to develop a simple model to predict and analyze the oxygen demand, diffusion, and concentration across the melon flesh.

### Oxygen Demand Meets Oxygen Diffusion

Firstly, oxygen demand was calculated from estimates of the rates of biomass production at different depths within the mesocarp (see Materials and Methods). This was first performed by expressing biomass as a function of time for each of the five layers analyzed above (Supplemental Figure S1). Fitted polynomials were then integrated to obtain the rates of biomass production and respiration was finally estimated by using a fruit construction cost (process-based) model previously applied to other fleshy fruits, such as tomato, kiwi, peach and grape berry (Liu et al., 2007; Dai et al., 2010, and references therein). Respiratory activity was calculated using either a low or a high estimate of the maintenance-linked respiration (Supplemental Figure S2). Next, the oxygen concentration was modeled assuming that a steady state O_2_ concentration at any position within the mesocarp is reached when O_2_ delivery (diffusion) equals O_2_ consumption (respiration). The complete mathematical formulation of this problem has already been described by McElwain (1978) for spherical biological objects. Briefly, the melon was modeled as a homogeneous sphere (i.e., without gas phase in the intercellular space) whose radius varied from stage to stage. Moreover, no O_2_ diffusion constraint was supposed to occur outside the melon. In contrast, a constrained diffusion of O_2_ across the skin (i.e., *h* coefficient) and within the mesocarp (i.e., *D* coefficient) was considered. Finally, the O_2_ gradient was assumed to vanish at the inner limit of the mesocarp, at half of the melon radius. Even though the measured O_2_ concentrations within the mesocarp largely exceeded the half saturation constant of the COX, the later parameter was taken into account in our calculations, as in Armstrong and Beckett (2011).

**FIGURE 5:**
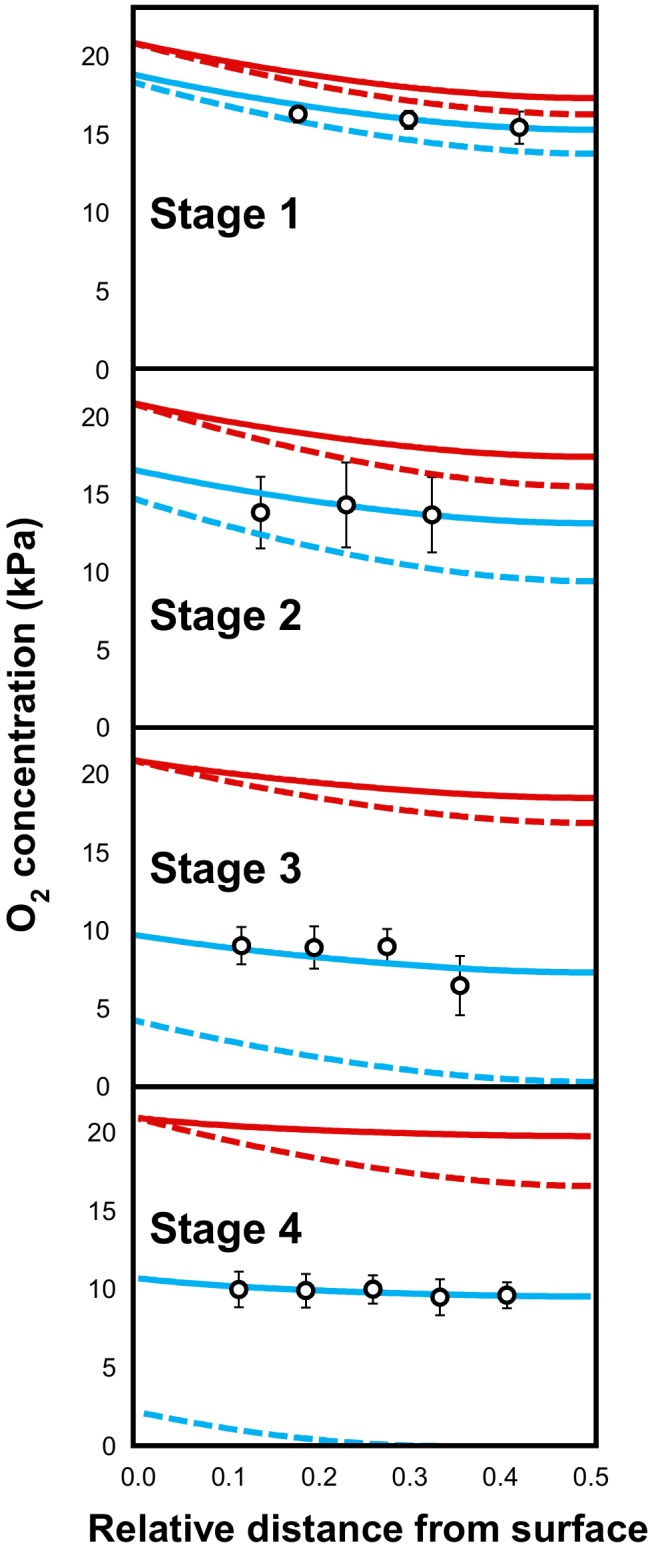
Comparison of measured (open circles) and calculated (red and blue unbroken or dashed lines) O_2_ tensions in the mesocarp of melon (*Cucumis melo* L. var Cantalupensis group Charentais cv. Escrito) fruit harvested at stages 1, 2, 3, and 4. O_2_ concentration was modeled assuming that a steady state O_2_ concentration is reached at any position within the mesocarp when O_2_ delivery by diffusion) equals O_2_ consumption by respiration. At each stage, a depth-dependent function of the respiratory activity was defined (see Supplemental Figure S2A) and the *h*-value (i.e., the permeability coefficient of the melon skin) was optimized to fit the measured O_2_ concentrations. Unbroken or dashed lines represent the O_2_ gradient profile when a gradient of respiratory activity within the tissue is present or not, respectively. Blue and red lines represent the O_2_ concentration within the tissue when a permeability barrier on the surface of the melon occurs (*h*-value is equal to 3.7 × 10^−5^, 1.4 × 10^−5^, 0.31 × 10^−5^, and 0.23 × 10^−5^ cm.s^−1^ at stages 1, 2, 3, and 4, respectively) or not (*h* = 10^−3^ cm.s^−1^), respectively. Oxygen concentration is expressed in kPa. A relative distance of 0 corresponds to the melon surface and 0.5, to the inner limit of the mesocarp.

Figure 5 represents the calculated and measured O_2_ tensions as a function of the position within the mesocarp and of the developmental stage. Several general trends can be drawn from the simulations. Firstly, the O_2_ gradient profile depends on the presence (unbroken line) or not (dashed line) of a gradient of respiratory activity within the tissue. Indeed, parameterizing the growth- and maintenance-dependent O_2_ consuming activity gradient with the COX enzymatic activity makes the O_2_ gradient flatter regardless of the melon stage. Secondly, the O_2_ concentration within the tissue depends on the external O_2_ availability, i.e., on the occurrence of diffusion constraints at the melon surface with (blue line) and without (red line) the permeability barrier on the surface of the melon). Indeed, setting a high *h*-value just makes the O_2_ concentrations higher within the tissue, the shape of the gradient being unchanged (Figure 5).

For each stage, the numerical solutions were compared with the corresponding analytical results (open circles in Figure 5). Good agreement between simulations and measurements were obtained when considering both a decrease in the O_2_ consumption rate from the surface to the center of the melon and a diffusion constraint at the surface of the melon. A least-square fit of the measured O_2_ concentrations gave the highest *h*-values in the earlier stages (3.7 10^−5^ and 1.4 10^−5^ cm.s^−1^ for melons harvested at stages 1 and 2) whereas this permeability coefficient value drastically decreased during later stages, to reach 0.23 10^−5^ cm.s^−1^ at stage 4 (Figure 5). The latter analysis was performed using a high estimate of the maintenance-linked respiration. It should be stressed that similar trends were observed with a low estimate (Supplemental Figure S4).

Even though respiratory activity is probably under the control of the major oxidase (i.e., COX), the modeling approach was repeated using the *K*_m_ value of alternative oxidase (AOX), which is one order of magnitude higher (0.134 vs. 0.0108 kPa, Armstrong and Beckett, 2011). It is worth mentioning that the O_2_ gradient profiles obtained using these values were very similar (Supplemental Figure S5). This tends to demonstrate that the affinity of the terminal oxidase of the respiratory chain does not greatly influence the balance between oxygen demand and diffusion throughout melon development (see also Armstrong and Beckett, 2011).

Overall, our modeling approach tends to demonstrate that the melon skin exerts increasing O_2_ diffusion constraints during fruit development which, in turn, may favor the establishment of hypoxia within the mesocarp tissue (see Supplemental Figure S4). However, the O_2_ consumption of this tissue undergoes a spatiotemporal decrease due to a concomitant decrease in the growth rate and the respiratory chain capacity. Therefore, melon development is characterized by a concomitant decrease in both the O_2_ demand and availability, thus ending with O_2_ partial pressure values above 40% regardless of the developmental stage and the section of the mesocarp.

### Integration of Metabolite Profiles, Enzyme Activities, and O_2_ Variables Reveals That the Cytochrome c Oxidase Capacity Is Tuned to Oxygen Availability

**FIGURE 6:**
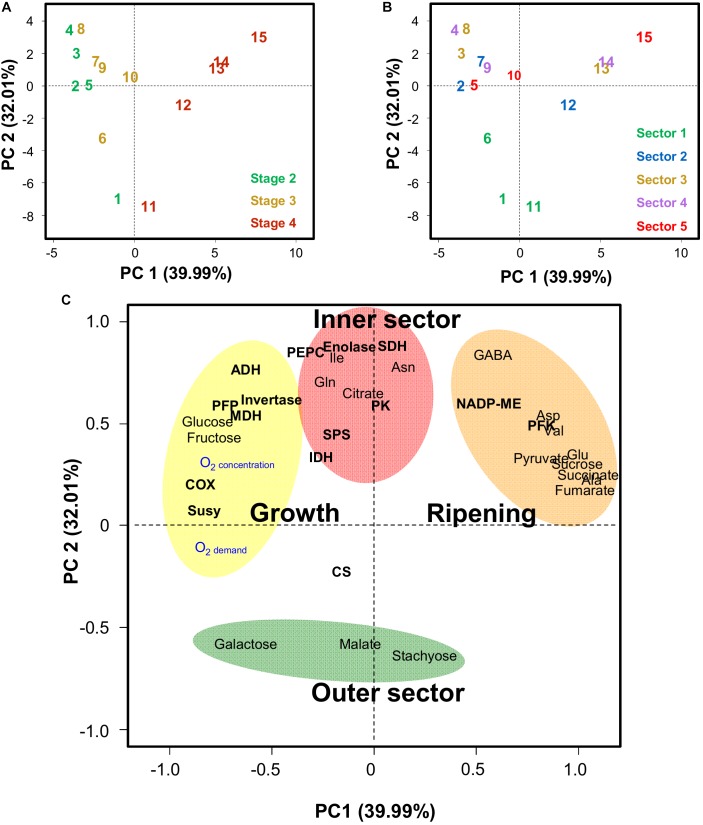
Principal component analysis (PCA) of estimated oxygen-demand and -concentration, 18 metabolites measured by quantitative ^1^H NMR spectroscopy and GC-EI-TOF/MS, and 15 enzyme capacities from central metabolism in five radial sections of the mesocarp (sector 1, to epicarp + green mesocarp; 5 inner orange mesocarp, see Figure 1) of melon (*Cucumis melo* L. var Cantalupensis group Charentais cv. Escrito) fruit at three stages of development, stages 1, 2, and 3. PCA was performed with averaged data expressed on a protein basis and on a FW basis (Supplemental Figure S6). **(A)** PCA scores plot of the first two principal components (PC1 and PC2) showing the distribution of the samples at three stages of development. **(B)** Same PCA scores plot of the first two principal components (PC1 and PC2) showing the distribution of the samples from the 5 radial sections of the mesocarp. **(C)** PCA loadings plot showing three different areas. Abbreviations for enzymes are given in the legend of Figure 3.

Principal component analysis (PCA) was used to integrate metabolite and enzyme data with O_2_ demand and concentrations (Figure 6). The data for oxygen concentration obtained with the fine glass microsensors did not correspond to the samples used for biochemical analysis and could therefore not be used directly to perform the PCA. Thus, the model described above was used instead, in order to obtain estimates of the oxygen concentration in the different sectors of the mesocarp and at the different developmental stages within which metabolites and enzymes had been measured. Finally, both O_2_ demand and O_2_ concentration estimates were included within the variables. The PCA was performed with averaged data of estimated oxygen-demand and -concentration, 18 metabolites measured by quantitative ^1^H NMR spectroscopy and GC-EI-TOF/MS, and 15 enzyme capacities from central metabolism in the five radial sections of the mesocarp expressed on a FW basis (Supplemental Figure S6) and on a protein basis (Figure 6). The latter gave the best separation of both samples and variables but the general trends were the same for both expression bases. The score plots (Figure 6A,B) indicate that the first principal component (PC1), which explains 40% of the total variance, separates the developmental stages whereas PC2, which explains 32% of the total variance, separates the sectors. The scores plots also indicate that the deeper the sector, the larger the amplitude of metabolic change over time. The corresponding loadings plot (Figure 6C) highlights four groups of variables, which were clearly associated to growth, ripening, the outer sector 1 (green outer mesocarp) and the inner sectors 2–5. The latter two groups of variables only responded weakly to development (low loadings along PC1). However, in the green outer mesocarp, galactose was closely associated with growth, suggesting that a decrease in carbon demand would lead to a small imbalance between the import and degradation of stachyose. The variable group attributed to growth was also associated with O_2_ demand and O_2_ concentration (Figure 6C). Strikingly, the latter two variables were well correlated with each other (*p* = 2.7 × 10^−5^) and with the capacities of the COX (*p* = 1.5 × 10^−3^ and 1.6 × 10^−6^, respectively) and to a lesser extent SuSy (*p* = 3.0 × 10^−2^ and 1.2 × 10^−2^, respectively). PFP was also significantly correlated to O_2_ concentration (*p* = 8.0 × 10^−4^). Glc and Fru, which were highly correlated with each other (*p* = 1.6 × 10^−12^), were also positively correlated (*p*-values ranging from 2.0 × 10^−2^ to 2.1 × 10^−6^) with the previous variables, whereas they were negatively correlated with Suc (*p*-values ranging from 5.9 × 10^−3^ to 1.4 × 10^−4^). Furthermore, Suc was strongly correlated with pyruvate, Ala, Glu, succinate and fumarate (*p* < 3.0 × 10^−4^). ME was also correlated to this group of metabolites. In contrast and as already seen above, changes in ADH were moderately associated to growth, and not to ripening, which implies that the rise in ADH was early during fruit development and not a response to the climacteric crisis.

### Tuning of Cytochrome c Oxidase Capacity to Oxygen Availability Is Not Under Transcriptional Control

Because of the strong correlation found between COX capacity on the one hand and O_2_ concentration or O_2_ demand on the other hand, the expression of genes coding for COX subunits was studied to check whether there is a transcriptional regulation in COX capacity in response to O_2_. Given the supramolecular organization of COX, subunits were selected according to their origin (nucleus vs. mitochondrial DNA encoded), their known function (catalysis vs. assembly) and their gene expression change – sometime in opposite directions – in response to oxygen availability (Mansilla et al., 2018). Finally, expression of COX1, COX5b, COX5c, COX6, COX11, and COX15 genes was studied by measuring the concentrations of the corresponding transcripts (Supplemental Figure S7). When transcripts were expressed on a protein basis, no significant correlations were found with COX capacity, O_2_ concentration or O_2_ demand. When transcripts were expressed on a FW basis, negative correlations were found between COX capacity and cox1 (*R*^2^ = 0.66, *p* = 2.5 × 10^−4^) as well as cox15 (*R*^2^ = 0.35, *p* = 2 × 10^−2^). O_2_ and O_2_-demand were also weakly and negatively correlated to cox1 (*R*^2^ = 0.34, *p* = 0.02, and *R*^2^ = 0.42, *p* = 0.009, respectively). Thus, the link found between COX capacity and O_2_ could not be attributed to an adjustment of the expression of these encoding genes.

## Discussion

### Metabolite Gradients Observed in Melon Fruit Reflect Metabolic Adjustments

The present study confirms that raffinose and stachyose, which are translocated via the phloem in the range of hundreds of mM (Haritatos et al., 1996; Knop et al., 2001), do not accumulate in the fruit flesh, thus indicating a rapid metabolism of these sugars within the fruit. The fact that stachyose and its degradation product galactose sharply decreased from the periphery to the inner mesocarp suggests that carbon import into the mesocarp is mostly centripetal. During fruit growth, glucose, fructose and to a lesser extent citrate were the most abundant metabolites found in the mesocarp. Contrary to stachyose and galactose, these metabolites were increasingly abundant toward the inner part of the mesocarp. This gradient may reflect a spatial difference in sugar metabolism that is perhaps linked to decreasing growth rate and thus decreasing carbon demand when going deeper into the mesocarp.

At ripening, the strong accumulation of a range of metabolites, i.e., sucrose, pyruvate, fumarate, succinate, alanine, glutamate, aspartate, asparagine, valine, and GABA (see also Moing et al., 2011), was probably enabled by the arrest of growth that occurred at around growth stage 3. Indeed, assuming that the import and processing of carbon and nitrogen would continue, a decrease in carbon demand would likely result in the accumulation of sugars and organic acids, and a decrease in protein synthesis resulting in the accumulation of amino acids. This accumulation also coincided with a relatively strong drop in the oxygen concentration (on average, from >60% to <50% of the atmospheric concentration), which was probably linked to a drop in the O_2_-permeability of the skin (Figure 5). Certainly, with the exception of fumarate, these metabolites, which are also precursors of a range of volatiles (Manriquez et al., 2006), are often accumulated in plant tissues under hypoxia (Menegus et al., 1989; Edwards et al., 1998; Roessner et al., 2001; Miyashita et al., 2007; Narsai et al., 2011). Among the enzymes studied here, only PFK and ME continued to accumulate during ripening (Figure 3, 6). Interestingly, the activation of ME under hypoxia has been shown to lead to the accumulation of pyruvate and Ala (Edwards et al., 1998). Besides, the metabolites that accumulated during ripening, were first seen to accumulate in the inner part of the fruit while oxygen gradients became flatter (they were more marked during growth stages 1–2), indicating that a more marked hypoxia was not the cause of their more pronounced accumulation. The fact that the capacities of most enzymes, in particular ADH and enzymes involved in glycolysis, were already higher in the inner part of the melon at the early growth stages could have potentiated such an accumulation, for example by favoring higher fluxes. Such “priming” phenomenon evokes the increased capacities of a range of enzymes involved in carbon metabolism (including PFP, PFK, Enolase, PK, and ADH), as observed in maize root tips pre-treated by hypoxia and that have been associated with improved survival under subsequent anoxia (Xia et al., 1995; Bouny and Saglio, 1996).

### Sucrose Accumulation Is Linked to Fermentation

The large increase in sucrose relative to glucose and fructose that is typically observed in melon has been attributed to the loss of soluble AI and the maintenance of SPS activity during ripening (Schaffer et al., 1987; Hubbard et al., 1989). However, in the present study AI activity was decreased at ripening but not lost, while SPS was maintained or even slightly decreased (Figure 3). This is actually in line with a recent study showing that whilst the mRNA of the only expressed gene encoding soluble invertase detected vanished in ripening melon fruits, invertase activity was still detected (Dai et al., 2011). The significantly negative correlation found between sucrose and SuSy suggests that decreasing SuSy may also be necessary for sucrose accumulation. Indeed, the highest SuSy activity was found in the outer mesocarp where sucrose, like stachyose, is probably unloaded from the phloem. Finally, the fact that sucrose was strongly correlated to a group of metabolites known to accumulate under hypoxia raises the question of a possible additional regulation of sucrose turnover by oxygen availability. In line with this, it has been reported that sucrose degradation is quickly inhibited in slices of potato tubers exposed to mild hypoxia (Geigenberger, 2003). Furthermore, in the roots and hypocotyl of soybean seedlings under hypoxia, sucrose has also been found to be accumulated despite maintained acid and alkaline invertase activities (Nanjo et al., 2010).

### Melon Fruit Achieves the Avoidance of Oxygen Gradient Within the Mesocarp

Oxygen gradients linked to oxygen diffusion have been reported for a range of plant systems. For example, potato tubers (Bologa et al., 2003) or soybean developing seeds (Borisjuk and Rolletschek, 2009) show dramatic O_2_ gradients when under optimal growth conditions, with the root nodules of legumes even achieving anoxic conditions within their center, an absolute requirement for N-fixation (Ott et al., 2005). Surprisingly, there were no such gradients in the mesocarp of melon fruit, even though hypoxia takes place and despite the fact that melons are relatively fast growing fruits that reach remarkably large sizes. A simple explanation is that the spherical form of the fruit guarantees that the O_2_ demand decreases from the outside to the inside, simply because there is considerably less biomass being produced in the inside than at the periphery. The model integrating O_2_ diffusion and demand presented here confirms this hypothesis.

Additionally, it is likely that mechanisms avoiding the waste of O_2_ taking place across the developmental stages studied here were also modulating the consumption of O_2_. Thus, SuSy and PFP, which were associated to the outer mesocarp where O_2_-demand was highest, are both considered as energy saving enzymes. The breakdown of Suc into hexose phosphates requires only one PPi when initiated via SuSy and two ATP when initiated via invertase (Geigenberger, 2003). Similarly, the phosphorylation of fructose-6P costs one PPi via PFP and one ATP via PFK, which again saves energy, especially in growing tissues where PPi is produced at high rates (Stitt, 1998). It is also striking that PFK activity increased quite dramatically at ripening, although O_2_-availability was decreased. It suggests that ATP-usage was no longer critical and/or that it was required to replace PPi-dependent reactions because PPi production had dropped, both events being consequences of arrested growth. This is in line with a recent study carried out on tomato pericarp which demonstrated that the energy-saving priority of fruit growth vanishes at the end of development thus leading to the onset of the climacteric crisis (Colombie et al., 2017).

Finally, the most remarkable metabolic adjustment to fluctuating O_2_ discovered by this study was the tuning of the capacity of COX to O_2_-availability. It is worth mentioning that such hypotheses are strongly debated in the literature (Armstrong and Beckett, 2011; Nikoloski and van Dongen, 2011). In plant cells, the local O_2_ tension results in a balance between oxygen diffusivity within the tissue and the activity of the oxygen consuming enzymes, especially COX, which is involved in the mitochondrial oxidative phosphorylation pathway. In dense, metabolically active and growing tissues such as seeds, seedlings, tubers and fleshy fruits, the lack of systems for O_2_ distribution leads to the limitation of oxygen diffusion, which may lead to a fall in the internal O_2_ concentrations (Borisjuk and Rolletschek, 2009). Admittedly, a falling internal O_2_ concentration results in a restriction of metabolic activity (Geigenberger, 2003). Within potato tubers (Geigenberger et al., 2000), pea and bean seeds (Rolletschek et al., 2002), it is paralleled by a severe decrease in the ATP/ADP ratio and adenylate energy charge (AEC), indicating that respiration is being inhibited. In melon fruits, despite a drastic decrease in ATP/ADP, from 31 (outer mesocarp) to 6 (inner mesocarp), the AEC is only slightly decreased, from 0.97 in the outer mesocarp to 0.83 in the inner mesocarp (Biais et al., 2009). As reported by Pradet and Raymond (1983), small variations in AEC with high values (between 0.85 and 0.95) imply significant variations in the ATP/ADP and ATP/AMP ratios and may thus conceal quite different regulatory situations for enzymatic ATP-utilizing processes. In a developing melon fruit, relatively high AEC and low ATP/ADP suggest that ATP-generating pathways (i.e., glycolysis and oxidative phosphorylation) are fitting the energy supply to the demands of the processes involved in growth and maintenance. Changes in both ATP/ADP and AEC have been observed *in vitro* and *in vivo* in potato tubers (Geigenberger et al., 2000) and tomato pericarp (Menu et al., 2004) under conditions of artificially low atmospheric oxygen pressure. Most importantly, the present study indicates that in melon fruit placed under atmospheric oxygen tension the decrease in the ATP/ADP ratio observed from the periphery to the inner mesocarp is not the consequence of an oxygen gradient within the tissue but rather a decrease in the capacity of the respiratory chain (i.e., the COX activity). Importantly, the affinity of COX for O_2_ corresponds to O_2_ concentrations that are much lower than those reported here, which suggests that regulatory events involving oxygen-sensing (Licausi et al., 2011; Weits et al., 2014) are taking place. The modulation of the capacity of COX therefore appears as a potent metabolic adjustment avoiding hypoxia. COX is usually considered as the rate-controlling step of the oxidative phosphorylation pathway. In various aerobically growing cells, down-regulation of COX activity participates to a fine-tuning of the respiratory capacity to changes in ATP demand, thus leading to energy status homeostasis (Devin et al., 2006). Here, our measurements provide evidence that the modulation of COX capacity (V_max_), rather than its apparent affinity for O_2_ (K_m_), can explain, at least in part, the spatiotemporal changes in the O_2_ concentration that are observed in bulky growing plant organs such as fleshy fruits. Transcriptional regulation is unlikely to occur in melon fruit since the transcript levels of nuclear- and mitochondria-encoded COX genes, especially COX5b and COX5c which are known to be downregulated by O_2_ deprivation in rice (Tsuji et al., 2000) sunflower (Curi et al., 2002) and Arabidopsis thaliana (Mansilla et al., 2018), were not positively correlated to the COX activity nor to the local O_2_ concentration. Therefore, mechanisms underlying the down-regulation of COX activity remain to be elucidated. However, it is clear that these changes do not result from a down-modulation of the mitochondria biogenesis *per se* (i.e., mitochondria content) since the evolution patterns of COX did not parallel with those of other mitochondrial enzymes (i.e., SDH, CS, and IDH). However, a modulation of the COX activity by means of post-translational modifications of regulatory subunits (Mansilla et al., 2018) is not to be excluded under our conditions.

### Skin as a Key Factor Controlling Hypoxia-Driven Maturation of the Melon Fruit

Oxygen deprivation is usually seen as leading to a serious disruption of plant metabolism, which is essentially an aerobic process (Geigenberger, 2003). As discussed above, melon fruit appears to be capable of maintaining a low-slope gradient of O_2_ concentrations throughout the mesocarp and at different developmental stages, especially during ripening where O_2_ concentrations were lowest (nearly 9 kPa corresponding to 40% of atmospheric O_2_). However, despite these mechanisms, the entire mesocarp became hypoxic quite early during fruit development (at stage 1, the average O_2_ concentration was already decreased by 24%). The modeling approach developed in the present study suggests that this was due to changes in skin permeability to O_2_. These computations are in line with gas transport measurements performed on pear and apple tissues showing that skin has a 20–50-fold lower O_2_ diffusibility than flesh (Ho et al., 2006, 2011). So, why would melon fruit build a skin that provokes hypoxia? Firstly, in many fruit species fermentative metabolic features are essential for the production of aroma volatiles during ripening (Manriquez et al., 2006). Hypoxia and/or products of fermentation such as ethanol and acetaldehyde are also triggers of fruit maturation (Pesis, 2005). Secondly, oxygen- and ethylene sensing mechanisms are known to interact at the molecular level within plants (Bailey-Serres et al., 2012), as well as fruits (Min et al., 2012), and oxygen promotes ripening in climacteric fruits by stimulating the ethylene response via a mechanism that obeys Michaelis–Menten kinetics (Beaudry, 1999). Therefore, decreasing internal O_2_ concentrations can also be seen as a way of controlling the rate of ripening.

## Conclusion

Taken together our results show that the entire mesocarp becomes hypoxic quite early during fruit development and suggest that in developing melon fruits O_2_ supply and O_2_ consumption are finely tuned by a combination of factors to ensure a timely maturation of the mesocarp. The spherical form of the fruit combined with the metabolic adjustments, especially the tuning of the capacity of COX to O_2_ availability that occurs during growth contribute to optimizing the O_2_ demand and avoiding the establishment of an O_2_ gradient within the flesh. The fact that such “controlled” hypoxia occurs relatively early in the fruit’s development would favor the increased activity of a range of enzymes involved in glycolysis and fermentation and that are essential for maturation. In addition the modeling approach developed in the present study suggests that the decrease in the skin permeability to O_2_ that coincides with the climacteric peak would moderate ripening while favoring fermentation throughout the mesocarp and almost at the same time, thus ensuring uniform maturation of the melon flesh. Skin permeability for oxygen therefore appears as a particularly interesting trait to investigate, especially in relation to metabolism, in further melon varieties and fruit species, as it could lead to improvements of fruit quality.

The assay for COX developed here is relatively easy to perform, thus providing an opportunity to investigate bulky organs in other species to verify whether COX adjustment to oxygen availability is a generic mechanism. The fact that the assay can also be performed in high throughput could then open a range of reverse and forward genetic approaches to investigate and manipulate hypoxia in plants. It will indeed be particularly interesting to try to investigate the regulation of respiration in relation to oxygen availability.

## Data Availability

The datasets for this manuscript are not publicly available because the data are provided in the Supplementary Data of the present manuscript. Requests to access the datasets should be directed to yves.gibon@inra.fr.

## Author Contributions

KM, BPB, DR, AM, and YG planned the experiments. KM, BPB, BB, MC, JA, CD, MM, RG, CC, and AM performed the experiments. KM, BPB, DR, and YG interpreted the data and wrote the manuscript. All authors have read and approved the manuscript.

## Conflict of Interest Statement

The authors declare that the research was conducted in the absence of any commercial or financial relationships that could be construed as a potential conflict of interest.

## Footnotes

1 http://faostat.fao.org/

2 http://primer3.ut.ee/

3 http://www.r-project.org/
