# Supplementary material for: Central Metabolism Is Tuned to the Availability of Oxygen in Developing Melon Fruit
Source: Front Plant Sci. 2019 May 15;10:594. doi: 10.3389/fpls.2019.00594 (PMC6529934; doi:10.3389/fpls.2019.00594)
Supplement: Supplementary file 1 [file Data_Sheet_1.pdf]

Supplementary Material

1 Supplementary Data

Supplemental Table S1: Raw data of metabolite concentrations and enzyme activities. Metabolites are expressed in µmol.g^{-1}FW, protein content in mg.g^{-1}FW and enzyme activities in nmol.g^{-1}FW.min^{-1} (means±SD; n=3)

**Stage**	**S2**	**S2**	**S2**	**S2**	**S2**	**S3**	**S3**	**S3**	**S3**	**S3**	**S4**	**S4**	**S4**	**S4**	**S4**
**Position**	**P1**	**P2**	**P3**	**P4**	**P5**	**P1**	**P2**	**P3**	**P4**	**P5**	**P1**	**P2**	**P3**	**P4**	**P5**
Alanine	0.2±0.02	0.19±0.03	0.22±0.06	0.27±0.15	0.27±0.15	0.41±0.04	0.73±0.16	0.96±0.76	1.4±1.49	1.79±2.02	2.82±0.61	10.2±0.17	16.43±1.37	18.44±0.71	19.54±0.09
Asparagine	0.12±0.01	0.14±0.02	0.15±0.01	0.15±0.01	0.14±0.01	0.23±0.04	0.18±0.04	0.18±0.02	0.16±0	0.16±0.04	0.1±0.03	0.19±0.02	0.24±0.03	0.25±0.02	0.31±0.05
Aspartate	0.53±0.03	0.65±0.07	0.78±0.07	0.88±0.12	1.08±0.16	0.78±0.07	1.24±0.32	1.4±0.47	1.39±0.51	2.17±1.09	1.89±0.21	2.61±0.22	2.48±0.14	3.1±0.2	4.78±0.65
Citrate	5.7±0.6	13.9±1.9	15.2±1.2	16.6±1	19.5±1.2	7.9±3.4	13.8±2.8	13.9±2.1	16.1±1.4	20.1±2.3	24.6±2.8	18.9±1	18.9±0.7	21.3±0.5	26.4±2.1
Fructose	85±4	105±6	112±2	123±3	110±2	82±3	97±6	101±2	110±3	97±2	109±9	89±11	82±4	85±4	67±2
Fumarate	0.04±0	0.01±0	0.01±0	0.01±0	0.02±0	0.03±0.01	0.02±0.01	0.02±0.01	0.03±0.03	0.04±0.02	0.09±0.01	0.2±0.06	0.25±0.05	0.29±0.03	0.37±0.06
GABA	0.39±0.03	0.75±0.04	0.85±0.01	0.91±0.05	0.91±0.1	0.59±0.12	0.9±0.1	1.04±0.12	1.12±0.21	1.27±0.41	0.73±0.04	1.53±0.12	1.83±0.18	2.04±0.13	2.7±0.14
Galactose	0.63±0.05	0.49±0.05	0.41±0.02	0.4±0.01	0.41±0.02	0.61±0.05	0.46±0.09	0.38±0.04	0.39±0.02	0.41±0.04	1.12±0.05	0.67±0.08	0.53±0.06	0.47±0.03	0.44±0.06
Glucose	72±3	98±7	107±2	119±2	107±2	65±1	86±5	95±1	107±2	93±4	58±6	62±8	64±4	70±5	53±4
Glutamate	0.58±0.04	0.5±0.04	0.57±0.09	0.63±0.1	0.65±0.15	0.85±0.04	0.83±0.2	0.97±0.45	1.18±0.83	1.42±1.02	2.08±0.17	3.43±0.46	4.16±0.41	5.14±0.53	6.79±0.78
Glutamine	3.7±0.8	5.2±1	5.2±0.4	5.2±0.5	3.2±0.5	7.6±1.4	7.1±1	7.2±0.6	5.8±0.1	4±0.9	2.6±0.8	6.4±1.1	9±1.9	8.2±1.5	7±1
Isoleucine	0.07±0.02	0.15±0.04	0.16±0.02	0.15±0.01	0.09±0.01	0.11±0.04	0.2±0.05	0.2±0.04	0.16±0.03	0.1±0.01	0.04±0.01	0.21±0.06	0.2±0.01	0.23±0.01	0.25±0.03
Malate	18.5±2	4.6±0.8	2.2±0.4	2.2±0.3	3.4±0.2	12.9±3	3.8±1.3	2±0.2	1.8±0	2.3±0.2	3.5±0.2	4.5±0.3	5.2±0.7	5±0.4	5.2±0.5
Phenylalanine	0.18±0.05	0.37±0.04	0.38±0.01	0.31±0.03	0.27±0.04	0.32±0.08	0.52±0.07	0.49±0.04	0.37±0.06	0.32±0.15	0.47±0.07	0.98±0.07	1.06±0.09	1.12±0.08	1.35±0.11
Pyruvate	0.03±0.01	0.02±0.01	0.03±0.01	0.03±0.01	0.02±0.01	0.04±0.01	0.02±0.01	0.03±0.01	0.02±0.01	0.04±0.01	0.06±0.02	0.06±0	0.1±0.05	0.06±0.03	0.13±0.04
Stachyose	0.1±0.01	0.08±0.05	0.07±0.04	0.04±0.02	0.08±0.01	0.16±0.04	0.08±0.03	0.08±0.03	0.09±0.05	0.14±0.08	0.38±0.1	0.16±0.04	0.12±0.02	0.13±0.04	0.14±0.02
Succinate	8.7±2.3	8.4±2.1	10.2±2.6	11.5±3.6	9.8±2	10.8±0.5	10.8±1.6	13.6±4.6	12.8±2.3	19.5±7.8	23.8±2.4	52.3±8.1	87.6±14.2	98.7±24.8	117.6±2.4
Sucrose	5.5±0.3	7.6±1.8	10.6±4.4	11.7±6.6	13.8±4.9	7.8±3	23.1±19.2	28.2±26.8	31.4±31.5	39±32.8	52.3±6.2	141±10	184.1±10.3	205±11.1	215.1±7
Tryptophan	0.032±0.007	0.043±0.011	0.041±0.004	0.023±0.008	0.022±0.012	0.063±0.024	0.074±0.028	0.056±0.011	0.038±0.017	0.045±0.024	0.108±0.022	0.184±0.025	0.199±0.043	0.215±0.032	0.325±0.026
Tyrosine	0.045±0.009	0.084±0.008	0.086±0.007	0.073±0.009	0.113±0.006	0.061±0.018	0.079±0.024	0.075±0.019	0.066±0.008	0.098±0.018	0.07±0.01	0.084±0.009	0.093±0.02	0.094±0.011	0.17±0.026
Valine	0.12±0.02	0.22±0.06	0.24±0.03	0.23±0.02	0.17±0.03	0.21±0.07	0.33±0.05	0.36±0.01	0.33±0.06	0.3±0.16	0.31±0.01	0.91±0.14	1.12±0.12	1.26±0.08	1.43±0.08
Protein content	3±0.3	2±0.2	1.9±0	1.8±0.1	2±0.1	2.7±0.1	2±0.1	2±0.3	2.3±0.7	2.3±0.3	4.1±0	3.2±0.1	3.2±0.1	3.3±0.1	3.4±0.2
ADH	3336±194	4347±473	5158±779	4819±315	4741±671	4307±247	4963±627	5130±410	4592±517	4451±362	3320±493	4163±653	5290±781	4991±386	5561±64
CS	38±1	34±6	20±5	24±4	30±8	40±4	29±3	31±1	26±2	34±2	59±4	48±3	43±8	51±2	35±1
Enolase	125±28	215±33	195±42	212±20	192±28	182±13	145±33	220±32	202±59	255±53	201±44	274±61	265±29	328±79	304±52
NADP-Malic Enz	39±4	28±3	27±9	28±7	30±5	42±15	39±7	33±9	30±8	47±12	51±7	52±9	62±8	59±12	69±18
IsocitDH	23±2	21±2	21±3	17±3	22±2	15±1	20±4	22±4	11±8	18±2	30±9	36±2	26±7	25±5	28±2
MDH	10748±1402	10973±1363	12366±1552	11317±1217	11813±1241	15072±761	12226±652	12231±56	11734±571	11332±911	13305±1859	11815±1550	13373±1877	13201±208	14317±265
PEPC	140±11	160±8	170±21	169±10	165±25	161±22	156±21	186±33	178±26	181±37	169±16	272±22	237±32	228±29	229±7
PFP	601±51	596±79	603±218	629±48	701±122	841±200	573±110	653±39	587±124	599±142	551±197	607±79	621±132	712±200	785±177
PFK	263±37	316±26	381±74	459±125	400±106	325±51	302±17	325±84	394±36	464±159	802±124	673±126	965±164	890±152	1287±107
PK	163±30	164±13	167±9	130±25	76±24	169±28	205±59	169±23	198±21	166±32	215±7	248±9	264±15	273±32	234±21
SPS	262±75	225±29	236±26	478±158	578±94	710±87	446±159	510±166	360±113	496±125	575±97	462±98	488±182	525±111	790±157
Susy	120±10	125±39	114±28	95±31	86±19	135±10	99±27	130±32	116±23	91±8	196±13	108±38	108±12	95±22	80±4
SDH	71±2	73±0	68±2	64±4	67±6	66±1	68±1	64±7	59±2	59±3	80±18	84±16	105±18	134±40	116±27
COX	1009±87	781±8	805±85	829±205	901±79	688±83	686±16	574±10	610±60	672±98	549±87	254±29	283±62	288±60	357±134
Acid invertase	102±9	296±129	670±221	695±561	69±0	298±0	360±62	1073±714	257±182	373±246	138±118	121±126	206±131	184±97	287±11

Supplemental Table S2: List of primers used in the study.

Gene name	Accession number	Forward Primer (5' > 3')	Reverse Primer (5' > 3')	Amplicon size
CmCOX 5b-2	MELO3C012937 T1	GTTTCTTTTGCAGTTCC TTCAGC	AGATGAGTTGCCAGTGT CCG	80
CmCOX 5C	MELO3C015922 T1	ACCTCTTTGTATTGCTC TTGGA	ACCACCAAAATCTGTCT CACTCA	93
CmCOX 6b	MELO3C011044 T1	TCCTGGTTTACATACAT GCCTAA	GGTCTTACAGGAGTGAT TCTAGC	82
CmCOX 11	MELO3C017589 T1	TGGACGGCATCAACAA TTTG	GATCTCTGGACCCTGTTC GG	90
CmCOX 15	MELO3C016120 T1	AAGTCGGATGCATCAG TTGA	CCTCCCCAACATCCAAC ACT	101
mtCOX1	EU069547.1	GGTTGTTGCCACCAAGT CTC	GATAGACCGTCCACCCA GTG	81
Spike 1	-	GATGCCCGACCATCATT TAG	GTGAGGGTAATGTCGCG TTC	75
Spike 2	-	ATTGCGCTCGCCATATA CAC	GCTGGGATCAGGAGGAG AAG	100
Spike 3	-	GATCGTTTGCCTGCATT ACC	GAGAGCGTCAGCCATAC CAC	103
Spike 5	-	ACAAAGGCAGCGTTGA AAAC	TAGTGTCTGCACGCCAT ACC	132
Spike 6	-	CGCAAAGTCTCTCCTCT TGG	CAGTAGCCATTGCGGAA GAT	115

2 Supplementary Note and Figures

```
function pde
t=linspace(0,100000000,100);
m=2;
b=1;
a=b*0.5; % melon mesocarp depth
x=linspace(a,b,100);
sol=pdepe(m,@pdex4pde,@pdex4ic,@pdex4bc,x,t);
u1=sol(:,:,1);
save('essaiCOX','x','u1');
figure(1)
plot(x,u1(end,:));
hold on;
title('Melon O2 gradient')
xlabel('normalized distance R')
ylabel('normalized O2 pressure C')
%----------
function [c,f,s]=pdex4pde(x,~,u,DuDx)
f=DuDx;
J=3.0458*x^3 - 6.4967*x^2 + 4.5497*x - 0.8632; % depth-dependent respiratory activity of 8 day-aged
melon (stage 1), in µmol O2.min-1.mL-1
% J=3.3572*x^3 - 7.1794*x^2 + 5.0421*x - 1.0566; % at 11 DAA (stage 2)
% J=1.246*x^3 - 2.5232*x^2 + 1.6703*x - 0.3145; % at 20 DAA (stage 3)
% J=0.9357*x^3 - 1.6007*x^2 + 0.888*x - 0.1415; % at 34 DAA (stage 4)
R=4; % melon radius at 8 dpa, in cm
% R=5.2; % at 11 DAA
% R=6.5; % at 20 DAA
% R=6.6; % at 34 DAA
P=21; % O2 pressure in water, in kPa
D=2.4*10^-5; % O2 diffusion coefficient at 25°C, in water cm2.sec-1
c=1/D;
Km=0.0108; % O2 affinity of COX, in kPa
% Km=0.134; % O2 affinity of AOX, in kPa
F=-J/60/1000/D*R*R*u(1)/(Km/P+u(1));
s=F;
%----------------------------------------------------
function u0=pdex4ic(~) % create initial conditions
u0=0;
%----------------------------------------------------
function [pl,ql,pr,qr]=pdex4bc(~,~,~,ur,~) % create boundary conditions%
h=3.7*10^-5; % O2 permeability coefficient of melon skin at 8 DAA, in cm.sec-1
% h=1.4*10^-5; % at 11 DAA
% h=0.31*10^-5; % at 20 DAA
% h=0.23*10^-5; % at 34 DAA
% h=10^-3; % no permeability constraint
D=2.4*10^-5;
R=4;
% R=5.2;
% R=6.5;
% R=6.6;
pl=0;
ql=1;
pr=h/D*R*(ur-1);
qr=1;
```

Note 1. Matlab script for modelling of the oxygen gradient within the melon mesocarp

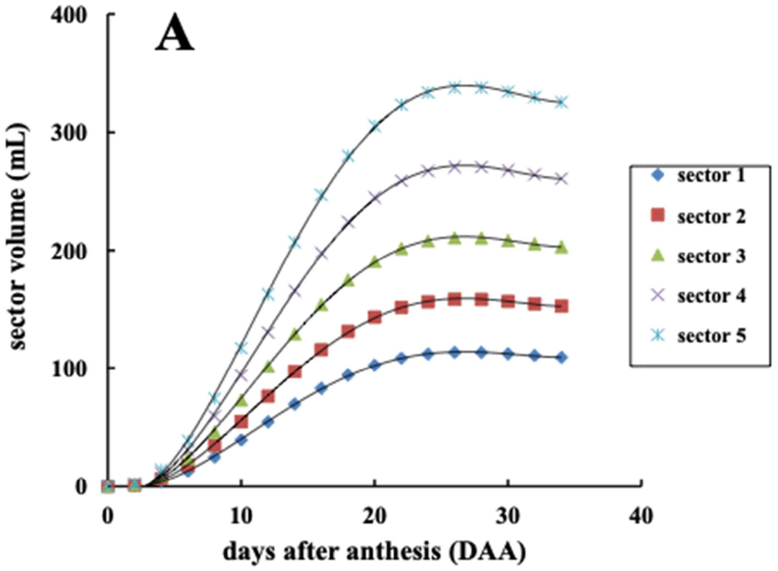

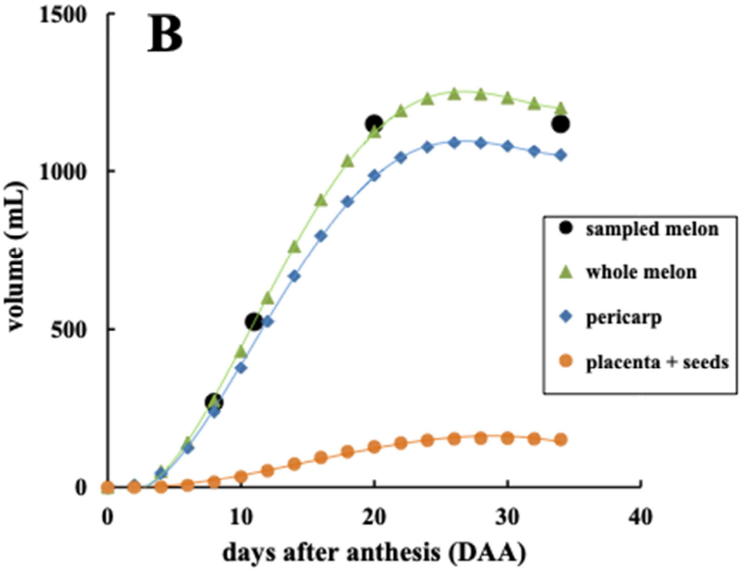

Supplemental Figure S1. Modelling melon fruit growth. (A) Time-dependent evolution of the pericarp sector volumes corresponding to the rings 1 to 5 of Fig. 2.(B) Time-dependent evolution of the inner part of the melon (placenta + seeds), of the pericarp (all sectors of panel A) and of the whole fruit. For comparison, the values corresponding to the melons sampled at stages 1 (8 DAA), 2 (11 DAA), 3 (20 DAA) and 4 (34 DAA) are superimposed. The density of the melon tissues was assumed to be equal to 1 g FW ml^{-1}.

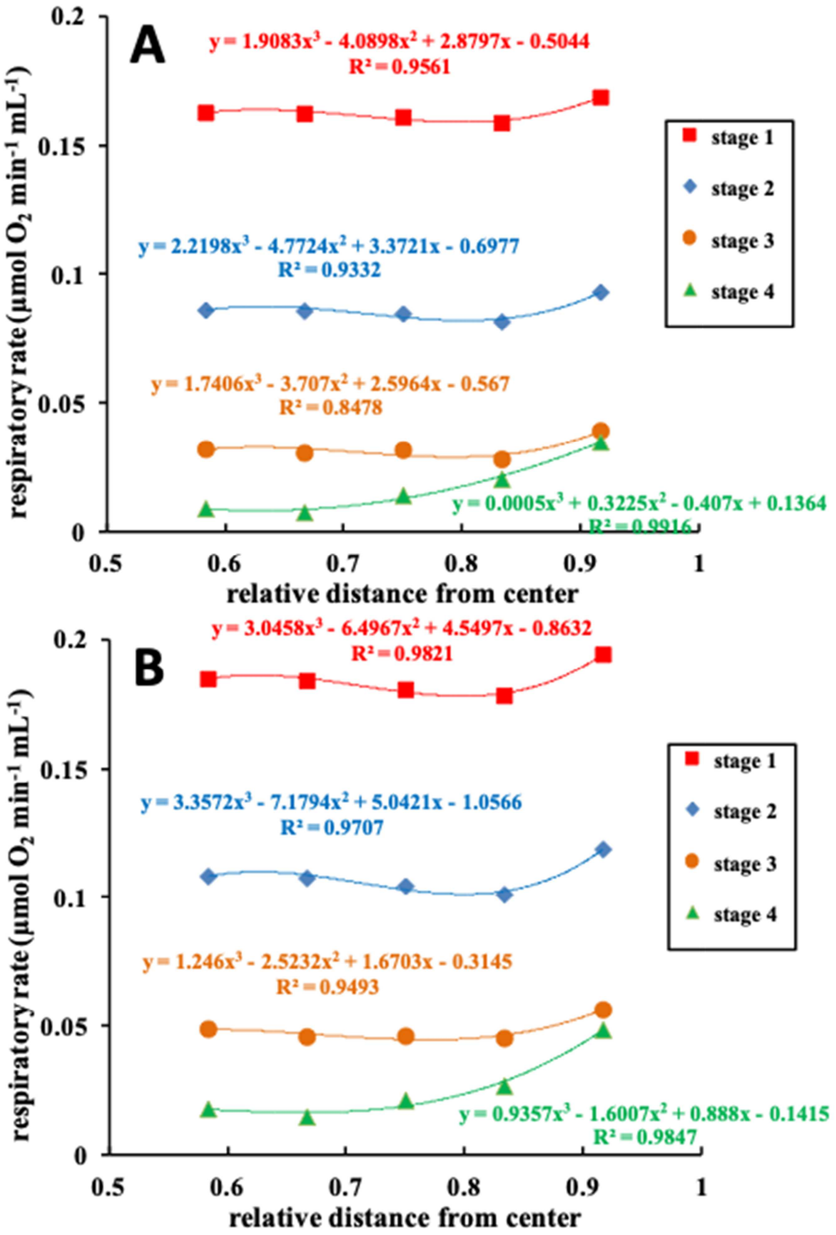

Supplemental Figure S2. Depth-dependent respiratory activity of melon pericarp as a function of developmental stage. Respiratory rate was calculated for each pericarp sector and each melon stage using the construction cost model. Briefly, the amount of oxygen consumed for growth was calculated taking into account the growth rate of the pericarp sector derived from supplemental Fig. 1A and assuming that the synthesis of 1 g of dry biomass needs the oxidation of 0.1 g of C. The amount of oxygen consumed for cell maintenance was estimated assuming that the maintenance-linked respiration of aerobic cells is proportional to the amount of respiratory chain complexes and is equal to 10% (A) or 20% (B) of the measured COX activity (see Materials and Methods). The continuous curves and the corresponding equations result from regression analyses of the data. A relative distance of 1 corresponds to the melon surface and 0.5, to the inner limit of the pericarp.

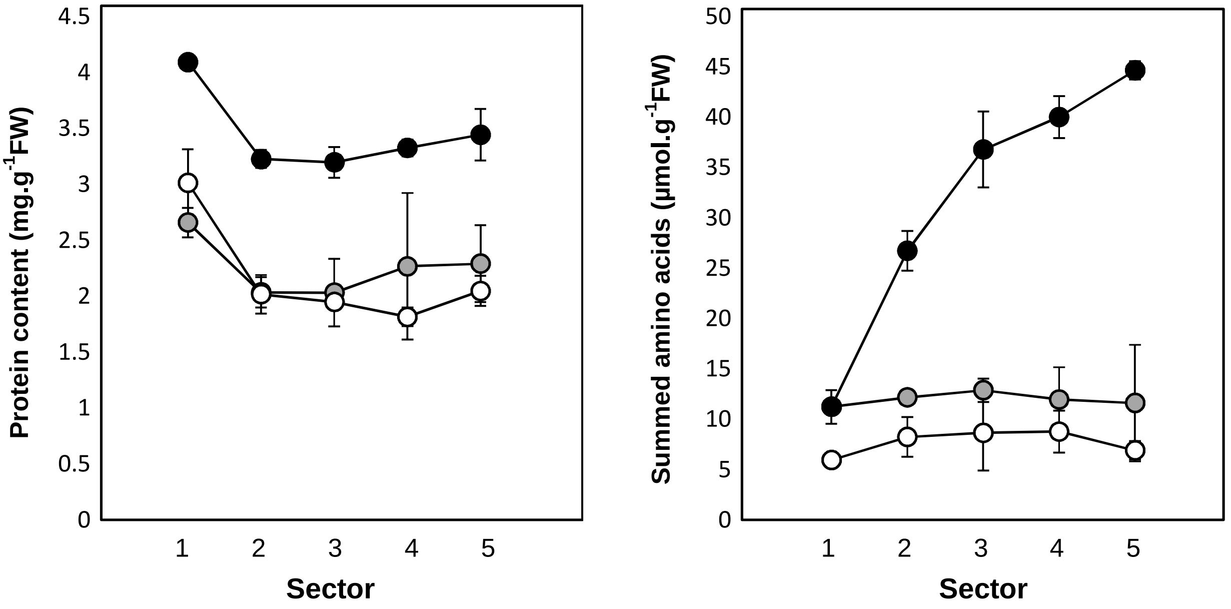

Supplemental Figure S3. Protein content and summed amino acids measured by quantitative ^{1}H NMR spectroscopy in developing melon fruit (*Cucumis melo* L. var. Cantalupensis group Charentais cv. Escrito) harvested at 3 developmental stages. The sector numbers 1, 2, 3, 4 and 5 correspond to five concentric mesocarp rings taken from the periphery (outer epicarp + green mesocarp, named sector 1) to the fruit centre (inner mescocarp, named sector 5). The results are the means of 9 measurements (3 biological replicates x 3 technical replicates), bars represent standard error (n=3). The data are available in Supplemental Table S1.

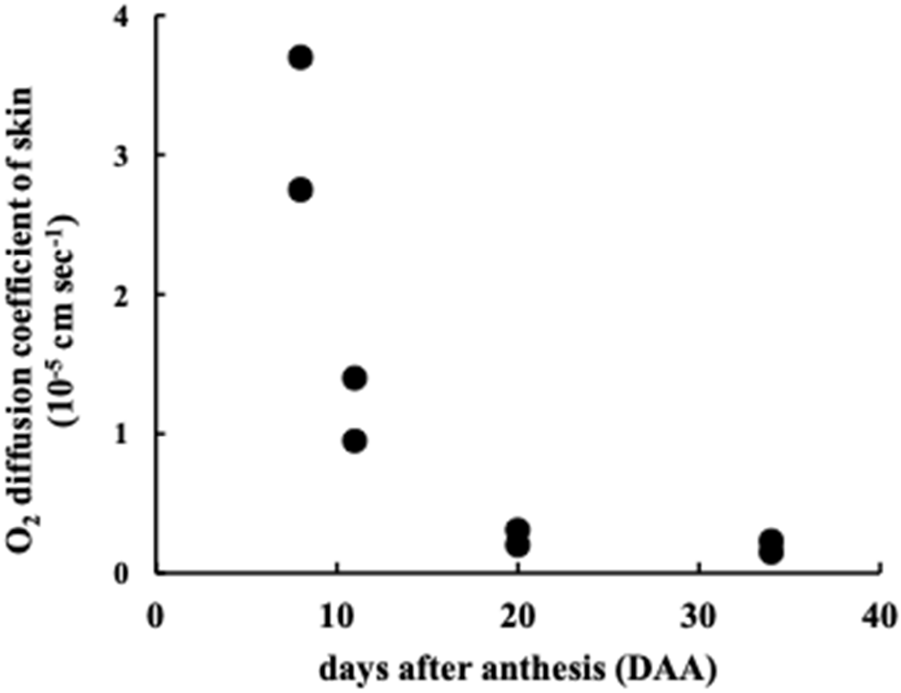

Supplemental Figure S4. O_2 permeability of melon skin as a function of developmental stage. The *h* values were calculated by least square fits of the O_2 gradient profiles as described in Fig. 5 and supplemental Fig. S3. The symbols represent two computations using low and high estimates of the maintenance-linked respiration according to supplemental Fig. S2-A and B, respectively.

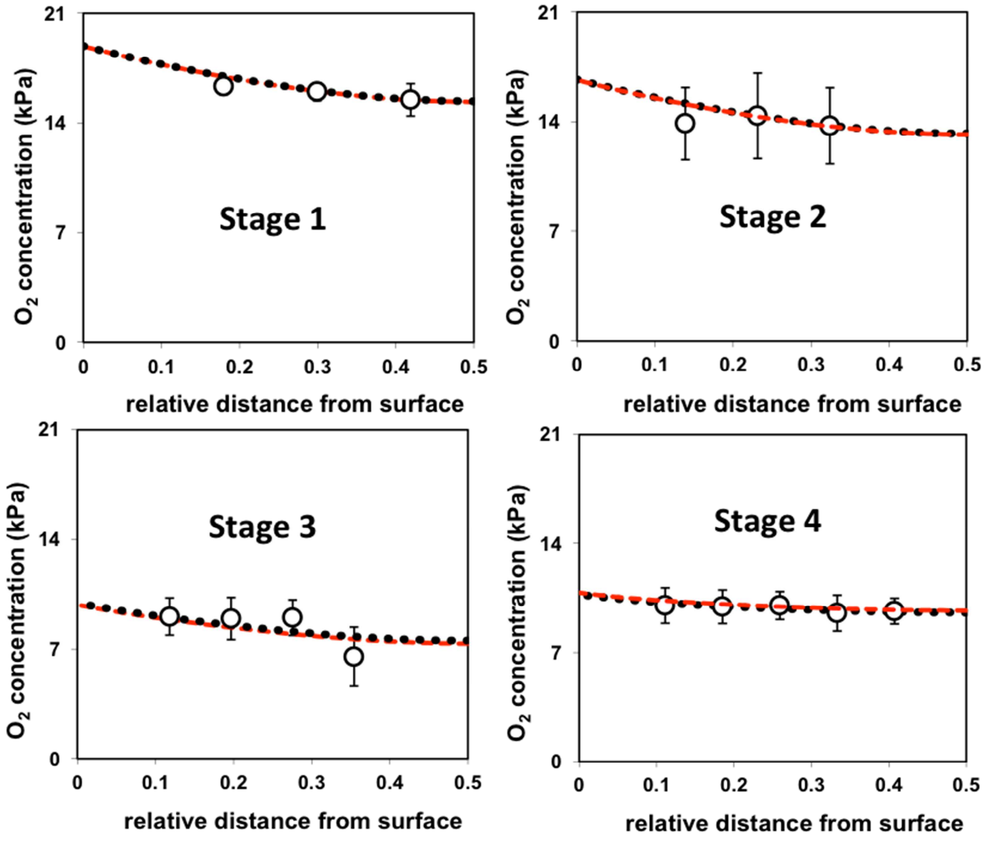

Supplemental Figure S5. Effect of O_2 affinity of terminal oxidases on the modelled O_2 gradients. Open symbols represent the O_2 tensions measured in the mesocarp of melon fruits harvested at stages 1, 2, 3 and 4. O_2 concentration was modelled as in Fig. 5 using either the K_m value of the COX (black dashed line) or that of the alternative oxidase (red dashed line). A relative distance of 0 corresponds to the melon surface and 0.5, to the inner limit of the pericarp.

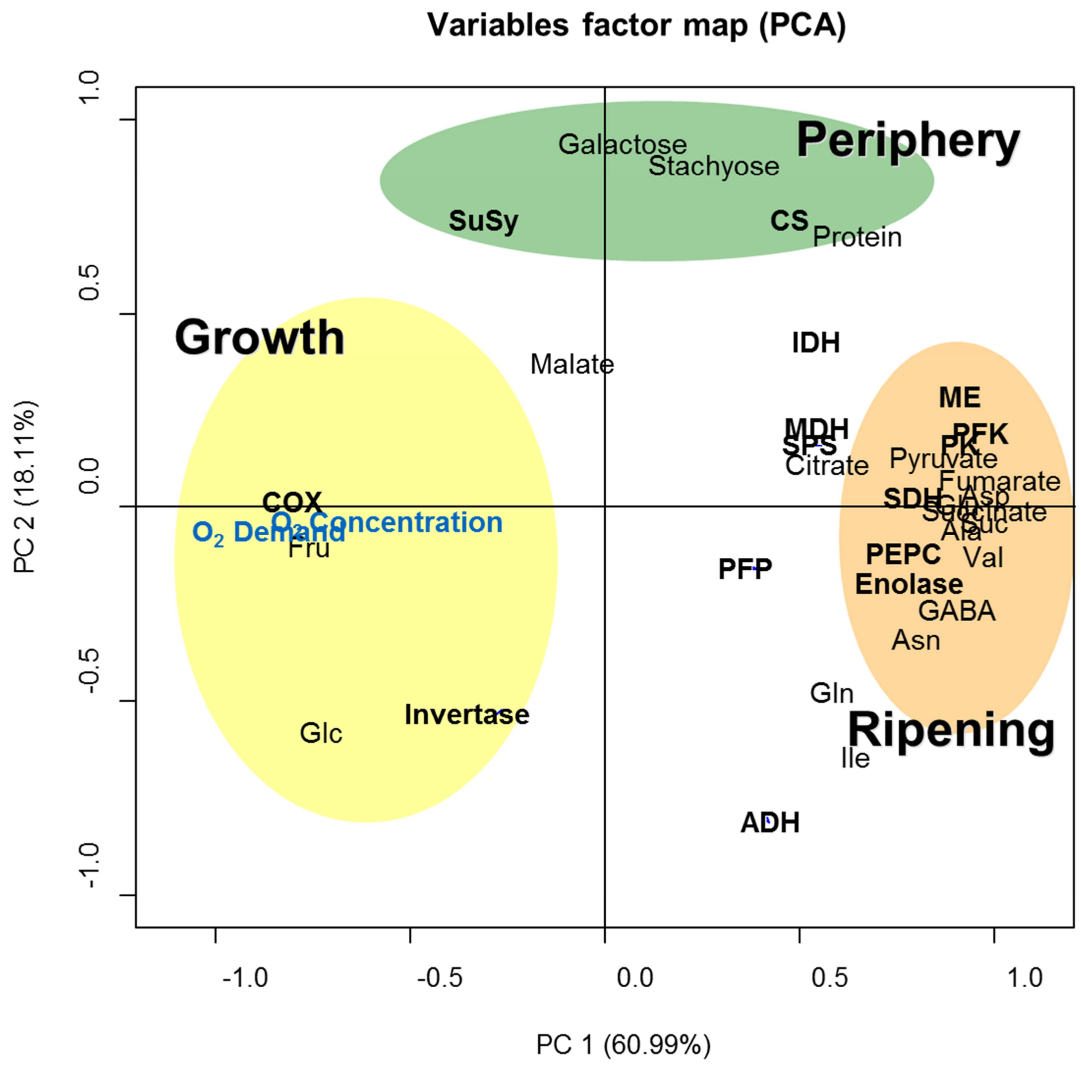

Supplemental Figure S6. Integration of metabolite profiles, enzyme activities, and O_2 variables expressed on a fresh weight basis. Principal component analysis (PCA) of estimated oxygen-demand and -concentration, 18 metabolites measured by quantitative ^1H NMR spectroscopy and GC-EI-TOF/MS, and 15 enzyme capacities from central metabolism in five radial sections of the mesocarp (sector 1, to epicarp + green mesocarp; 5 inner orange mesocarp, see Fig. 1) at three stages of development, stages 1, 2 and 3. PCA loadings plot showing three different areas. The PCA was performed with averaged data expressed on a fresh weight basis. Abbreviations for enzymes are given in the legend of Fig. 3.

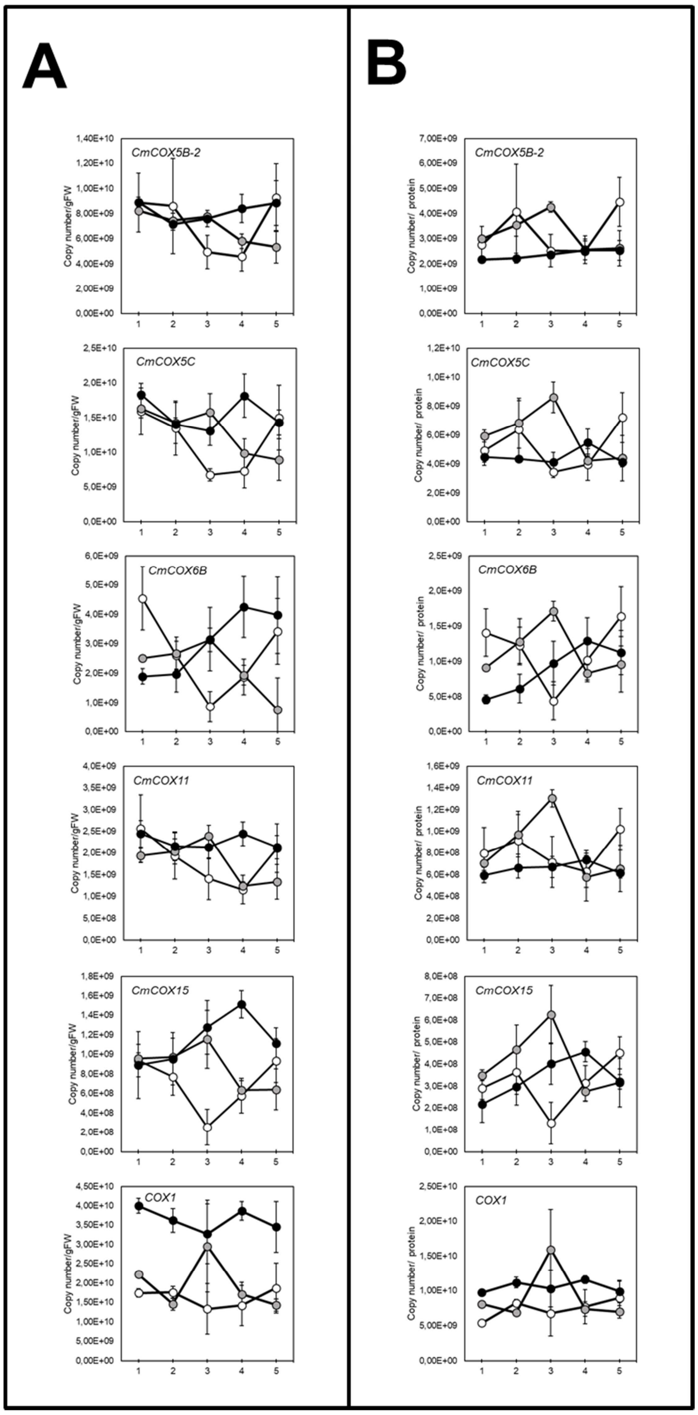

Supplemental Figure S7.Cytochrome c oxidase gene expression during the development of melon (*Cucumis melo* L. Escrito cv) fruit harvested at 3 developmental stages. Concentrations of mRNA encoding subunits COX1, COX5B-2, COX5C, COX6, CO11 and COX15 of the respiratory complex IV were measured by qRT-PCR. Sectors 1, 2, 3, 4 and 5 correspond to five concentric mesocarp rings taken from the periphery (outer epicarp + green mesocarp, named sector 1) to the fruit centre (inner mescocarp, named sector 5). Transcript concentrations are expressed as (A) copy number per gram fresh weight or (B) copy number per g protein.
